# DNA binding by the Rad9A subunit of the Rad9-Rad1-Hus1 complex

**DOI:** 10.1371/journal.pone.0272645

**Published:** 2022-08-08

**Authors:** Bor-Jang Hwang, Rex Gonzales, Sage Corzine, Emilee Stenson, Lakshmi Pidugu, A-Lien Lu

**Affiliations:** 1 Department of Biochemistry and Molecular Biology, University of Maryland School of Medicine, Baltimore, Maryland, United States of America; 2 Marlene and Stewart Greenebaum Comprehensive Cancer Center, University of Maryland School of Medicine, Baltimore, Maryland, United States of America; 3 Nathan Schnaper Internship Program in Translational Cancer Research, University of Maryland Medical Center, Baltimore, Maryland, United States of America; Cornell University, UNITED STATES

## Abstract

The Rad9-Rad1-Hus1 checkpoint clamp activates the DNA damage response and promotes DNA repair. DNA loading on the central channel of the Rad9-Rad1-Hus1 complex is required to execute its biological functions. Because Rad9A has the highest DNA affinity among the three subunits, we determined the domains and functional residues of human Rad9A that are critical for DNA interaction. The N-terminal globular domain (residues 1–133) had 3.7-fold better DNA binding affinity than the C-terminal globular domain (residues 134–266) of Rad9A^1-266^. Rad9A^1-266^ binds DNA 16-, 60-, and 30-fold better than Rad9A^1-133^, Rad9A^134-266^, and Rad9A^94-266^, respectively, indicating that different regions cooperatively contribute to DNA binding. We show that basic residues including K11, K15, R22, K78, K220, and R223 are important for DNA binding. The reductions on DNA binding of Ala substituted mutants of these basic residues show synergistic effect and are dependent on their residential Rad9A deletion constructs. Interestingly, deletion of a loop (residues 160–163) of Rad9A^94-266^ weakens DNA binding activity by 4.1-fold as compared to wild-type (WT) Rad9A^94-266^. Cellular sensitivity to genotoxin of *rad9A* knockout cells is restored by expressing WT-Rad9A^full^. However, *rad9A* knockout cells expressing Rad9A mutants defective in DNA binding are more sensitive to H_2_O_2_ as compared to cells expressing WT-Rad9A^full^. Only the *rad9A* knockout cells expressing loop-deleted Rad9A mutant are more sensitive to hydroxyurea than cells expressing WT-Rad9A. In addition, Rad9A-DNA interaction is required for DNA damage signaling activation. Our results indicate that DNA association by Rad9A is critical for maintaining cell viability and checkpoint activation under stress.

## Introduction

In response to DNA damage or replication block, cell cycle checkpoints provide surveillance mechanisms to activate the DNA damage response (DDR), which in turn elicits both DNA repair processes and cell cycle arrest (allowing time for DNA repair) [1, 2]. If the DNA damage is too extreme, apoptosis is triggered. During DDR, the single-stranded DNA (ssDNA) binding protein (RPA) coats ssDNA and recruits the ATR-ATRIP (ataxia telangiectasia and Rad3-related protein and ATR interacting protein) complex [3, 4]. At the same time, the Rad9-Rad1-Hus1 (9-1-1) checkpoint clamp is loaded onto 5’-recessed DNA by Rad17-RFC_2-5_ [5–7] and interacts with topoisomerase IIβ-binding protein 1 (TopBP1) to activate ATR [8–11] which then phosphorylates hundreds of downstream proteins including Chk1 kinase [4, 12–14]. Most mammalian genomes encode two different Rad9 proteins, Rad9A and Rad9B [15, 16]. In addition to its role in DDR [8, 17], 9-1-1 is directly involved in many DNA transactions including base excision repair (BER) (reviewed in [18–20]). It has been suggested that 9-1-1 provides a platform to coordinate BER processes because it interacts with and stimulates nearly every enzyme in BER [19]. Rad9A can also act independently to regulate expression of a limited set of target genes [21–23]. The biological significance of 9-1-1 is demonstrated by abnormal DDR, genomic instability, and embryonic lethality with the deletions of any of its subunits [18, 24–28]. For example, *mRad9A* knockout (KO) mice are embryonic lethal, and mice with targeted deletion of Rad9A in skin keratinocytes are susceptible for skin tumors [24, 29]. Interestingly, the Rad9A protein can function as an oncogene or tumor suppressor as both aberrantly high or low Rad9A expression have been linked to breast, lung, thyroid, skin, and prostate tumorigenesis [30–32]. Because tumor cells rely on DNA repair and DDR for proliferation and survival, these pathways are attractive targets for novel anticancer drugs [33, 34].

**Fig 1:**
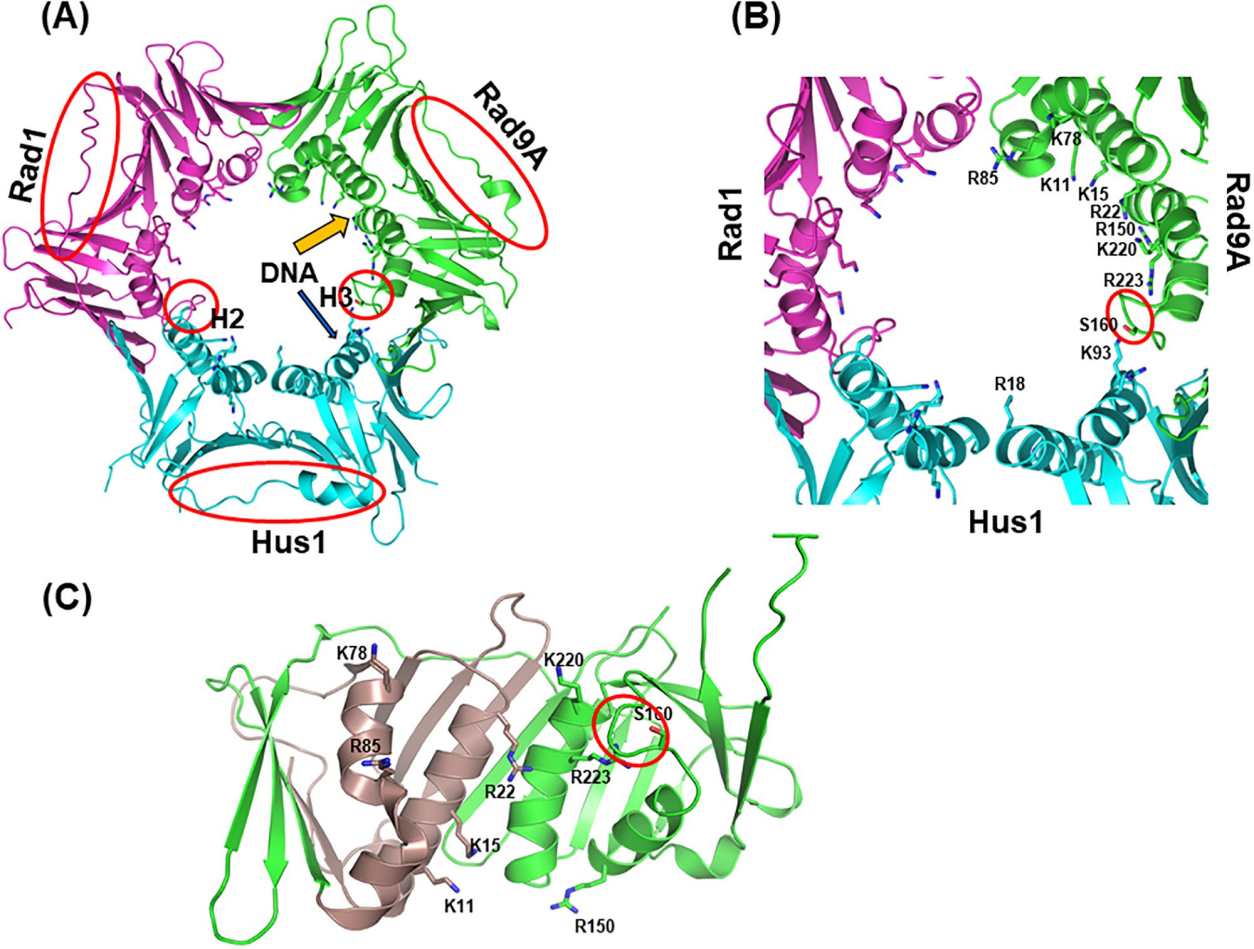
Structure of the 9-1-1 complex. (**A**) Top view of the human 9-1-1 complex. The crystal structure 9^1−272^-1-1 is shown (RCSB codes:3A1J) [37]. The extreme C-terminal domain of Rad9A (residues 267–391) is not shown because its structure has not been determined. The key differences among the three subunits are circled in red. The DNA in the inner channel of the 9-1-1 ring is bound by Rad9A and the N-terminal domain of Hus1 [43] shown in orange and blue arrows, respectively. (**B**) Basic residues and S160 of hRad9A as well as R18 and K93 of hHus1 in the channel of the 9-1-1 ring are labeled. A small loop (residues 160–163, with sequence SPAL) of hRad9A projected into the inner ring surface is circled in red. (**C**) A side view of hRad9A structure looking from inside of the ring. Residues 1–93 are colored dark violet and residues 94–266 are colored green. A small loop (residues 160–163) is circled in red.

The ring structure of 9-1-1 heterotrimer [35–37] is remarkably similar to that of proliferating cell nuclear antigen (PCNA) [38, 39]. Like PCNA, the N- and C-terminal globular domains of each subunit of 9-1-1 are connected by unique interdomain connecting loops (IDCLs). The three subunits of 9-1-1 are structurally similar but exhibit key differences (Fig 1A). These differences are most pronounced in their IDCLs and an extra C-terminal tail on Rad9A [35–37]. This structural asymmetry correlates with an asymmetry in protein-protein interactions and DNA binding. For example, the Hus1 subunit preferentially binds MYH (MUTYH) DNA glycosylase and apurinic/apyrimidinic endonuclease (APE1) [40–42] while Rad1 has high affinity to FEN1 [35]. Both human 9^1−272^-1-1 and 9^1−266^-1-1 (lacking the unstructured Rad9A C-terminal tail) can bind DNA [37, 43]. We have shown that the individual subunits of 9-1-1 have different DNA binding affinities with the order of strongest to weakest Rad9A^1-266^ > Hus1 >> Rad1 [43]. Intriguingly, the affinity of Rad9A^1-266^ subunit with blunt ended DNA is comparable to that of the 9^1−266^-1-1 complex [43]. However, 9^1−266^-1-1 has a much stronger affinity to 5’-recessed DNA substrates than Rad9A^1-266^ subunit [43]. These biochemical studies have prompted us to hypothesize that individual subunits of 9-1-1 play differential roles in DDR and DNA repair.

Like PCNA homotrimer, 9-1-1 heterotrimer is thought to interact with the DNA phosphate backbone, affording proper loading at sites of DNA damage and scaffolding activity on chromatin [44, 45]. According to the structure of 9-1-1 alone [35–37] and PCNA-DNA complex [46, 47], basic residues on the inner ring surface of each subunit are predicted to bind DNA (Fig 1B). However, their direct interactions have not been demonstrated. It has been shown that mutations of multiple positively charged residues in the inner ring of mouse Hus1 (mHus1) cause defects in chromatin localization and ATR signaling activation [48]. Similarly, basic residues K11, K15, R22, K78, R85, R150, K220, and R223 on the inner ring surface of Rad9A (Fig 1B and 1C) have been predicted to be in close proximity to DNA [35–37]. Because Rad9A has the highest DNA affinity and Rad1 has the weakest DNA affinity among the 9-1-1 subunits [43], we investigated the role of the basic residues of human Rad9A (hRad9A) on DNA binding. We also generated a loop-deletion (LD) mutant of hRad9A by deleting residues 160–163 (with sequence SPAL) which project into the channel of the ring [35–37] (red circle in Fig 1B and 1C). Analyses of domain deletions and point mutations demonstrate that basic residues in the inner ring at both N- and C-terminal domains and a small loop of hRad9A synergistically contribute to DNA binding. We also examined the biological defects of hRad9A mutants. Cellular sensitivity to hydroxyurea (HU, a DNA replication blocker) of *rad9A* KO cells is restored by expressing WT-Rad9A^full^, R150A/R223A double mutant (DM) Rad9A^full^, and K220A Rad9A^full^, but not by expressing LD-Rad9A^full^ mutant. Cellular sensitivity to H_2_O_2_ of *rad9A* KO cells is restored by expressing WT-Rad9A^full^, but not by expressing R150A/R223A double mutant (DM) Rad9A^full^, LD-Rad9A^full^, and K220A Rad9A^full^ mutants. DDR activation as measured by Chk1 phosphorylation requires Rad9A association with DNA. Our results indicate that defects in DNA binding of Rad9A render cells sensitive to oxidative stress and diminished checkpoint activation.

## Materials and methods

### Cell culture

*rad9A*^+/+^ (wild type, WT) and *rad9A*^-/-^ human prostate cancer PC3 cells [49, 50] (obtained from Dr. Howard Lieberman at Columbia University) were maintained in RPMI 1640 supplemented with 15% fetal bovine serum, 100 I.U./ml penicillin, and 100 μg/ml streptomycin.

### Plasmids

Plasmid pETDuet-hRad9A^1−266^ has been described [43]. pACYCDuet-hRad9A^1−266^ was constructed by digesting pETDuet-hRad9A^1−266^ with SalI and BamHI and the Rad9A fragment was ligated with SalI and BamHI-digested pACYCduet-1 (Millipore-Sigma). Plasmid p3XFLAG-CMV-14 (Sigma-Aldrich) containing *Rad9A* cDNA (FLAG-hRad9A) [51] was obtained from Dr. Mihoko Kai at Johns Hopkins University.

Genes encoding His-tagged hRad9A^1−133^, hRad9A^134−266^, and hRad9A^94-266^ were cloned by PCR method using pETDuet-hRad9A^1−266^ [43] as template and primers listed in S1 Table. The PCR products were digested with SalI and BamHI, then ligated into SalI and BamHI digested pACYCDuet-1. The mutant *hRad9A*^*1-266*^ genes encoding His-tagged proteins containing K11A/K15A/R22A (TM), K11A/K15A/R22A/K78A (QM1), R150A, R223A, and R150A/R223A (DM mutant), and Δ160–163 (LD mutant) were constructed by the QuikChange Site-Directed Mutagenesis kit (Stratagene) using pETDuet-hRad9A^1-266^ as the template. Genes encoding proteins for His-tagged Rad9A^94-266^ proteins containing K220A, R150A, R223A, R150A/R223A (DM mutant), and Rad9A^94-266^ Δ160–163 (LD mutant) were subcloned as described for WT-hRad9A^94-266^ except using respective pETDuet-hRad9A^1-266^ mutants as the templates.

To express FLAG-tagged hRad9A mutants in mammalian cells, full-length mutant *Rad9A* genes were cloned into p3XFlag-CMV 14.1 using Pro Ligation-Free Cloning Kit (Applied Biological Materials Inc. Canada). Briefly, the mutant *Rad9A* genes in pACYCDuet-1 were PCR amplified with primers (Rad9A 11-223-F and Rad9A 11-223-R) and the products were incubated with linearized plasmid p3XFLAG-CMV-14-hRad9A [51] (PCR amplified with Linearize-F and Linearize-R as listed in S1 Table) in master mix at 50°C for 30 min according to manufacturer’s procedures. The overlapped ends of the two DNA molecules allow for recombination. The PCR primers and mutagenesis oligonucleotides are listed in S1 Table. All the hRad9A clones were confirmed by DNA sequencing.

### Protein purification from *E*. *coli* cells

His-tagged hRad9A^1−266^ (wild-type and mutants) were purified from *E*. *coli* BL21Star/DE3 harboring pETDuet-hRad9A^1−266^ over Ni-NTA resin and 1 ml Hitrap SP (GE Healthcare) as described [43]. hRad9A^1−133^ (wild-type and TM mutant), hRad9A^134−266^, hRad9A^94−266^ (wild-type and mutants) were purified similarly as hRad9A^1−266^ except that they were expressed in *E*. *coli* LOBSTR-BL21(DE3) (Kerafast, EC1002) and purified using 5 ml hydroxyapatite CHT column (Bio-Rad) and 1 ml Hitrap SP column after Ni-NTA resin. The fractions containing respective proteins were pooled, concentrated, and buffer exchanged by Amicon (Millipore-Sigma). The samples were then divided into small aliquots and stored at –80°C.

### Dynamic light scattering (DLS)

Solubility analysis of all proteins were carried out by DLS using SpectroLight 600 (XtalConcepts GmbH, Hamburg, Germany). Terasaki microbatch plate (Nunclon Delta; catalogue No. 1–36528, Nunc GmbH, Wiesbaden, Germany) was covered with a thin layer of mineral oil to protect the solutions from drying out. Protein sample (1 μl) was then dispensed into the wells under oil. A DLS-laser beam with a wavelength of 660 nM at a power of 100 mW was focused on the drop and the scattered light is measured by the detector kept at an angle of 150°. All samples were measured at room temp. The poly dispersity index (PDI) was calculated as the weighted average molecular weight divided by the number average molecular weight. Proteins with PDI values < 30% were considered as soluble.

### Electrophoresis mobility shift assay (EMSA DNA binding assay)

The DNA binding activities of Rad9A and its mutants were assayed as previously described [43]. The binding reactions (20 μl) contained 20 mM Tris–HCl, pH 7.6, 80 mM NaCl, 1 mM DTT, 1 mM EDTA, 1.5% glycerol, 200 ng/ml poly dI/dC, 50 μg/ml bovine serum albumin, 10 nM 5’-fluorecein-tagged HC40/HG40 DNA (see S1 Table), and various concentrations of proteins. After electrophoresis on a nondenaturing 6% polyacrylamide gel in 50 mM Tris-borate, pH 8.3, and 1 mM EDTA buffer, the gel fluorescence images were detected using a Typhoon FLA9500 (GE Healthcare). Enzyme-bound and free DNA bands were quantified by ImageQuant Total software (GE Healthcare).

### Western blotting

For analysis of 9-1-1 induced DDR, *rad9A*^-/-^ human PC3 cells were transiently transfected with FLAG-hRad9A [51] or FLAG-Rad9A mutants using X’tremeGENE HP transfection reagents (Millipore-Sigma). The control *rad9A*^+/+^ and *rad9A*^-/-^ PC3 cells were treated with 16 mM HU for 2 hours and recovered for 2 hours or left untreated. Cells from one 10 cm dish (~1 × 10^7^cells) were lysed in 0.3–0.5 ml of RIPA buffer (50 mM Tris–HCl, pH 7.4, 150 mM NaCl, 1% NP40, 1 mM EDTA, 0.1% TritonX-100, 1 mM phenylmethylsulfonyl fluoride, 1 mM NaF, and 1 mM Na_3_VO_4_) at 4°C for 30 min followed by centrifugation at 14,000 rpm for 10 min. The supernatant was aliquoted and stored at −80°C. The protein concentration was determined by Bio-Rad protein assay (Bio-Rad). Proteins were fractionated using 10% SDS-polyacrylamide gels and transferred to a nitrocellulose membrane. The membranes were allowed to react with antibodies against phosphorylated Chk1 (pChk1) at Ser317 (S317) (Cell Signaling), total Chk1 (Bethyl Laboratories) or β-actin (Sigma). Western blotting was detected by the Enhanced Chemiluminescence (ECL) analysis system (GE Health) according to the manufacturer’s protocol. Signals were detected by GE-Amersham Imager 680 RGB.

The expression levels of Rad9 protein in *rad9* KO cell were also analyzed by Western blotting as described above. Total Rad9A was detected by unphosphorylated monoclonal antibody #14484 (Rad9A (D2J4P) from Cell signaling Technology.

### Cell viability analysis

FLAG-Rad9A and FLAG-Rad9A mutant plasmids were transfected into *rad9A*^-/-^ PC3 cells and cells were examined for genotoxin sensitivity as described [52]. Cells (about 1,000 cells) were seeded in 6-well culture plates. One day post-seeding, the cells were transfected with plasmid using X’tremeGENE HP transfection reagents. Cells then were selected by 125 mg/ml G418 for stable lines. Non-transfected *rad9A*^+/+^, *rad9A*^-/-^ PC3, and transfected cell lines were then seeded in 12-well culture plates (about 2,500 cells/well). One day post-seeding, cells were treated with 2 mM HU for 2 hrs or 80 μM H_2_O_2_ for 1 hrs in serum-free medium. Untreated cells were used as controls. Cells were then washed with PBS and fresh medium was replaced. After recovery for 72 hrs, the plates were incubated for 2 hrs in regular medium containing 40 μg/ml of neutral red (3-amino-7-dimethylamino-2-methyl-phenazine hydrochloride, Sigma). The cells were then washed with PBS twice; the dye was extracted from each well with acidified ethanol solution. Plates were incubated at room temperature with gentle shaking for 15 minutes and the absorbance at 540 nm was read in Multiskan Spectrum microplate spectrometer (Thermo Scientific).

## Results

### Determination of the DNA binding domains of Rad9A

**Fig 2:**
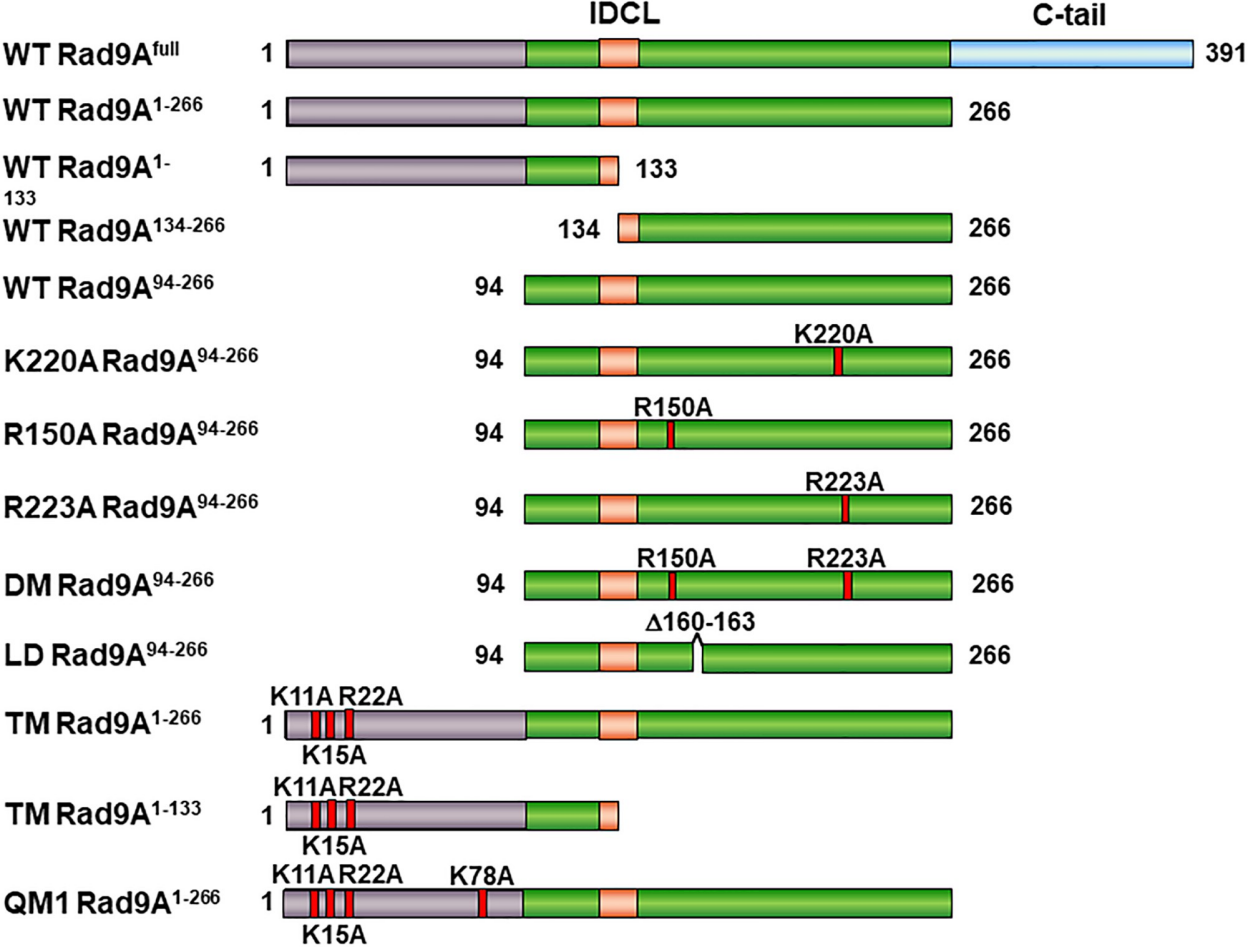
The hRad9A constructs and mutants used in DNA binding assay are depicted. Numbers represent residues of hRad9A. Residues 1–93 are colored dark violet and residues 94–266 are colored green except residues 125–140 (colored pink) which are the interdomain connecting loop (IDCL) between the N- and C-terminal globular domains. The C-terminal tail (C-tail) (colored blue) of hRad9A is not required for DNA binding.

**Fig 3:**
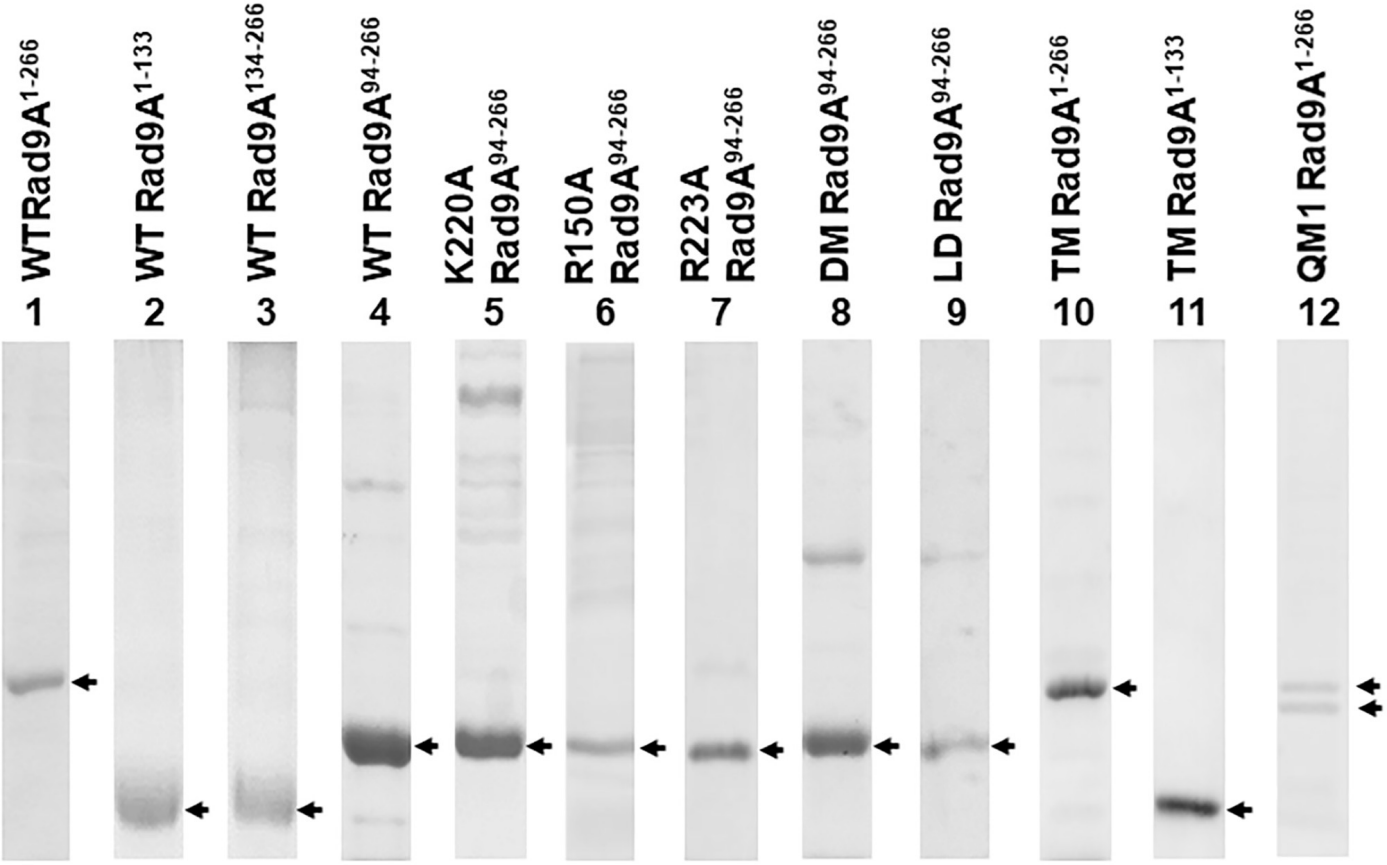
Purified His-tagged hRad9A constructs. Proteins were analyzed by 12% -15% SDS-polyacrylamide gel electrophoresis and stained by Coomassie Blue. Corresponding protein bands are indicated by arrows.

**Fig 4:**
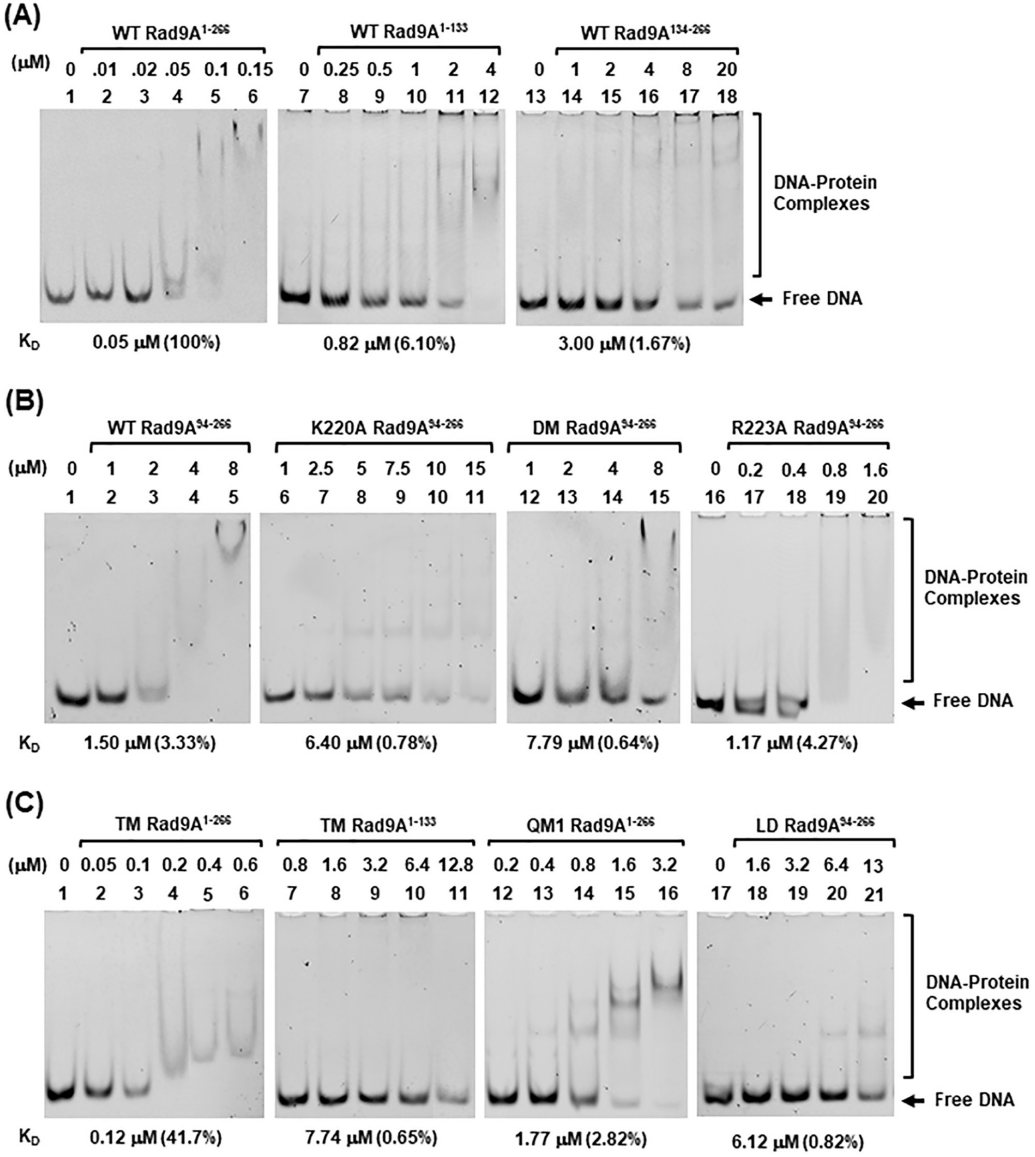
DNA binding activities of hRad9A mutants. Binding with blunt-ended HC40:HG40-DNA was performed by EMSA. The protein:DNA complexes were fractionated on 6% non-denature polyacrylamide gels. (**A**) DNA binding of wild-type (WT)-hRad9A deletion constructs. (**B**) DNA binding of Rad9A^94-266^ constructs containing mutations. DM represents R150A/R223A double mutant. (**C**) DNA binding of different Rad9A constructs containing mutations. TM, QM1, and LD represent K11A/K15A/R22A, K11A/K15A/R22A/K78A, and loop deletion (Δ160–163) mutants, respectively. Protein concentrations are indicated above each lane. Enzyme-bound and free DNA bands were quantified by ImageQuant Total software (GE Healthcare) to calculate the percentage of DNA binding. Based on the EMSA data, the Rad9A protein concentration resulting in a 50% of band shift was estimated as K_D_. The K_D_ values and relative DNA binding are indicated below each panel. Free DNA substrate is marked by an arrow and the protein:DNA complexes including shifted discrete bands or smear regions are marked.

Previously, we have shown that the individual subunits of 9-1-1 can bind DNA and have different DNA binding affinities [43]. Because Rad9A subunit has the highest DNA affinity among 9-1-1 subunits [43], we analyzed which domains of Rad9A are involved in DNA binding. Human Rad9A consists of 391 residues which includes an extended C-terminal tail (residues 267–391) (Fig 2). It has been shown that the C-terminal tail of Rad9A is not required for and may have a negative effect on DNA binding of the 9-1-1 core ring [37, 43, 53]. Therefore, we divided tail-less core hRad9A into two halves (Rad9A^1-133^ and Rad9A^134-266^), each containing one globular domain with two α-helices and 50% of the IDCL [35–37] (Fig 2). These two hRad9A halves were expressed as N-terminal His-tagged protein in *E*. *coli*, purified to over 90% purity (Fig 3, lanes 2 and 3), and tested for binding with blunt-ended 34-mer DNA (HC40/HG40) by electrophoresis mobility shift assay (EMSA). As shown in Fig 4A (lanes 7–18), each half formed specific protein-DNA complexes. Based on the EMSA data, the Rad9A protein concentration which leads to a 50% of band shift was estimated as K_D_. Table 1 shows the dissociation constants (K_D_s) and the relative DNA binding affinities of Rad9A^1-266^. Rad9A^1-133^ had 3.7-fold better DNA binding affinity than Rad9A^134-266^, however, the DNA binding affinities of Rad9A^1-133^ and Rad9A^134-266^ were 16- and 60-fold weaker than that of Rad9A^1-266^, respectively (Table 1). Thus, although both halves of hRad9A contribute to DNA binding, their binding affinities are substantially weaker than intact core hRad9A.

**Table 1:** Dissociation constants (Kd) and relative DNA binding affinities for Rad9A deletion and point mutants.

Construct	K_d_ (μM)	Relative DNA Binding
**WT Rad9A** ^**1-266**^	**0.05 ± 0.01**	**100**
**WT Rad9A** ^**1-133**^	**0.82 ± 0.15**	**6.10 (*100*)**
**WT Rad9A** ^**134-266**^	**3.00 ± 0.62**	**1.67**
**WT Rad9A** ^**94-266**^	**1.50 ± 0.40**	**3.33 (** **100** **)**
**K220A Rad9A** ^**94-266**^	**6.40 ± 0.75**	**0.78 (** **23.4** **)**
**R150A Rad9A** ^**94-266**^	**Not detected**	-
**R223A Rad9A** ^**94-266**^	**1.17 ± 0.69**	**4.27 (** **128.2** **)**
**DM Rad9A** ^**94-266**^	**7.79 ± 0.88**	**0.64 (** **19.2** **)**
**LD Rad9A** ^**94-266**^	**6.12 ± 1.27**	**0.82 (** **24.6** **)**
**TM Rad9A** ^**1-266**^	**0.12 ± 0.03**	**41.7**
**TM Rad9A** ^**1-133**^	**7.74 ± 0.68**	**0.65 (*10*.*7*)**
**QM1 Rad9A** ^**1-266**^	**1.77 ± 0.79**	**2.82**

Based on the EMSA data (Fig 4), the Rad9A protein concentration which leads to 50% of band shift was estimated as K_d_. The results shown are the mean and standard deviation for three independent experiments. Relative DNA binding (fold) is calculated as compared to WT Rad9A^1-266^, WT Rad9A^94-266^ (shaded yellow and underlined), or WT Rad9A^1-133^ (shaded green and italicized).

During the purification of Rad9A^1-266^ protein, we observed some protein degradation. To avoid protein degradation, we determined the N-terminal sequence of one of the major degraded polypeptides through Edman degradation analysis by University of California at Davis Genome Center Core Facilities. The N-terminal sequence of this polypeptide is VEK which represents residues 94–96 of hRad9A. Thus, N-terminally truncated hRad9A^94-266^ protein was expressed and purified with reduced protein degradation (Fig 3, lane 4). EMSA showed that soluble Rad9A^94-266^ formed specific complexes with DNA (Fig 4B, lanes 1–5). Rad9A^94-266^ had a 30-fold weaker DNA binding affinity than Rad9A^1-266^, but bound DNA 2-fold better than Rad9A^134-266^ (Table 1), indicating that residues 1–93 (colored dark violet in Fig 1C) are important for DNA binding. These results indicate that both N- and C-terminal domains of hRad9A contribute to DNA binding and display binding synergy.

### The involvement of positively charged residues on the inner ring surface of Rad9A in DNA binding

The inner ring surface of the 9-1-1 complex is covered by multiple Lys and Arg residues [35–37] (Fig 1B) which have been suggested to interact with the DNA phosphate backbone [35]. Six positively charged mouse Hus1 (mHus1) residues (K25/K93/K173/R175/K236/K237) have been shown to synergistically facilitate mHus1 chromatin localization following genotoxic stress [48]. However, the involvement of these six residues of mHus1 in direct DNA binding has not been demonstrated. Eight basic residues (K11, K15, R22, K78, R85, R150, K220, and R223) on the inner ring surface of hRad9A (Fig 1B and 1C) have been predicted to be in close proximity to DNA [35]. Therefore, we mutated these Arg/Lys residues to Ala in different combinations within several hRad9A deletion constructs (Fig 2) and compared their DNA binding affinities.

First, we examined the three basic residues (R150, K220, and R223) on the C-terminal globular domain of hRad9A which is adjacent to the Hus1 subunit (Fig 1B). Purified Rad9A^94-266^ protein containing a K220A mutation (Fig 3, lane 5) had a 4.3-fold reduction in DNA binding affinity as compared to WT-Rad9A^94-266^ (Fig 4B, compare lanes 6–11 to lanes 2–5 and Table 1). Rad9A^94-266^ containing R150A, R223A, and R150A/R223A [double mutant (DM)] (Fig 2) were also purified (Fig 3, lanes 6–8) and examined by EMSA. Analyses indicated that R223A had 1.3-fold stronger binding affinity compared to WT- Rad9A^94-266^, while R150A/R223A (DM) mutant had 5.2-fold reduced DNA binding affinities (Fig 4B, compare lanes 12–20 to lanes 2–5, and Table 1). We could not detect any DNA binding activity with R150A Rad9A^94-266^ likely because it was insoluble as indicated by dynamic light scattering (DLS) analysis with polydispersity index (PDI) value of 44%. These results indicate that K220A and R150A/R223A (DM) mutants are defective in DNA binding.

There are five positively charged residues (K11, K15, R22, K78, and R85) on the inner ring surface of the N-terminal globular domain of hRad9A (Fig 1B and 1C). Numerous combinations of mutations would be required for systematical analyses of these residues. Therefore, we only constructed and tested three mutants. K11A/K15A/R22A triple mutant (TM) of Rad9A^1-266^ bound DNA 2.4-fold weaker than WT-Rad9A^1-266^ (compare lanes 2–6 of Fig 4C to lanes 2–6 of Fig 4A, and Table 1). However, triple mutant of Rad9A^1-133^ (TM-Rad9A^1-133^) reduced DNA binding by 9.3-fold as compared to WT-Rad9A^1-133^ (compare lanes 7–11 of Fig 4C to lanes 8–12 of Fig 4A, and Table 1). These data indicate that K11A/K15A/R22A triple mutations weaken DNA binding at a higher extent when they reside within a shorter hRad9A construct. One quadruple mutant, K11A/K15A/R22A/K78A (QM1) (Fig 2), was constructed for further analyses of DNA binding (Fig 4C, lanes 12–16). The QM1-Rad9A^1-266^ mutant had reduced DNA binding affinities by 35- and 14-fold as compared to WT-Rad9A^1-266^ and TM-Rad9A^1-266^ respectively (Table 1). These results suggest that K11, K15, R22, and/or K78 are important for binding to the DNA phosphate backbone.

### DNA binding of a loop-deleted Rad9A mutant

By inspecting the Rad9A structure within the 9-1-1 complex, we observed the existence of a small loop (residues 160–163 with sequence SPAL) which projects into the ring channel (circled in red in Fig 1). Although this loop does not contain basic residues, we suspect that it may have a role in DNA binding. Thus, we deleted this small loop of hRad9A to generate the LD-Rad9A^94-266^ mutant (Fig 2) and examined its DNA binding. As shown in Fig 4C (lanes 18–21), the binding affinity of LD-Rad9A^94-266^ was 4.1-fold weaker than that of WT-Rad9A^94-266^ (Table 1). This result indicates that the loop (residues 160–163) is critical for DNA binding.

### Rad9A mutants with defects in DNA binding are more sensitive to hydrogen peroxide

**Fig 5:**
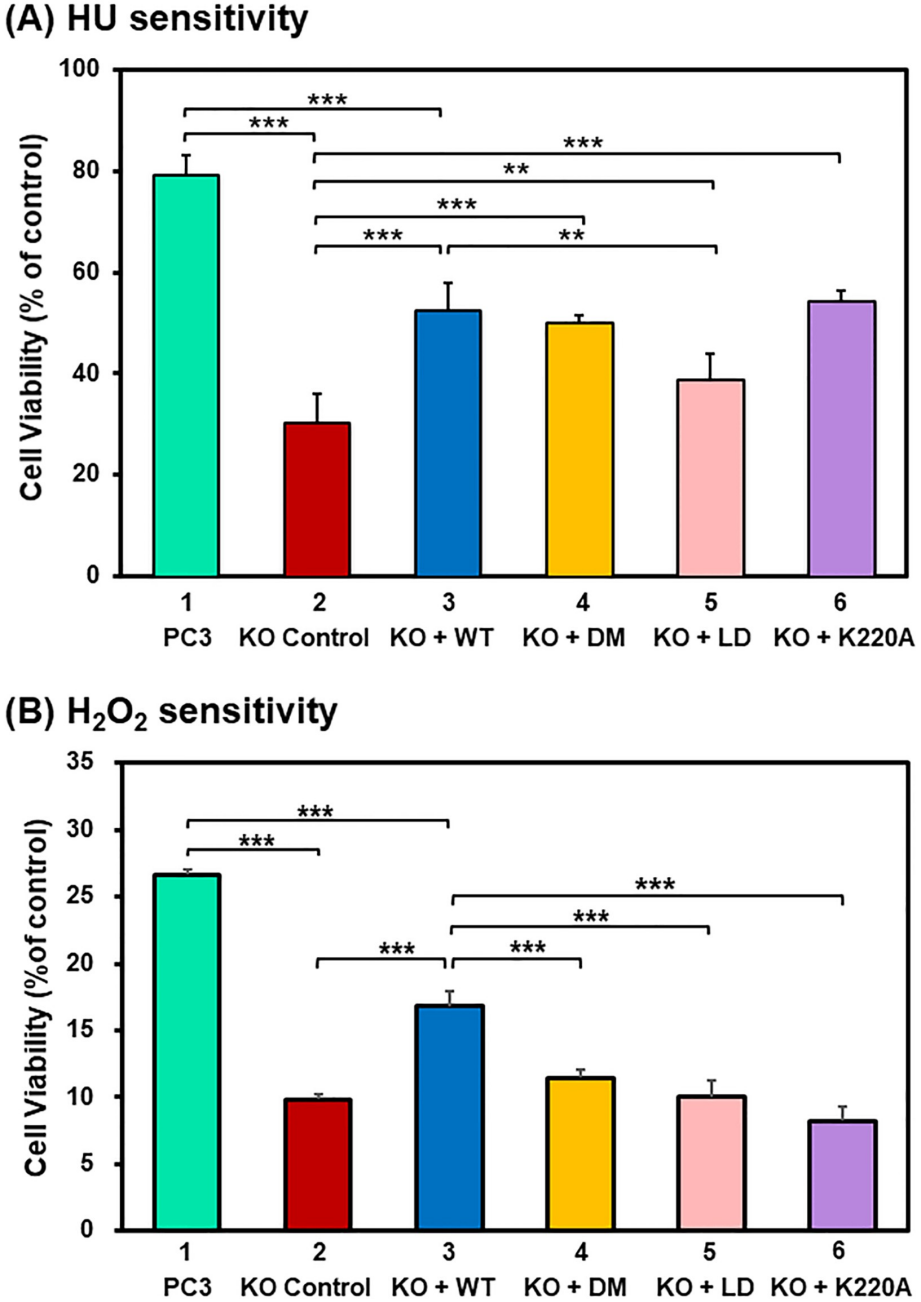
Cellular sensitivities to hydroxyurea (HU) and H_2_O_2_ of PC3 *rad9A*^+/+^ and *rad9A*^-/-^ cells expressing WT-Rad9A or mutant Rad9A. (**A**) Cell viability in response to hydroxyurea (HU). *rad9A* KO PC3 cells were transfected with vector p3XFLAG-CMV-14 (KO control) or vector containing *hRad9A* cDNA [(FLAG-WT-hRad9A, KO + WT), (FLAG-DM-Rad9A, KO + DM), (FLAG-LD-Rad9A, KO + LD) or (FLAG-K220A-Rad9A, KO + K220A)]. Plated cells were treated with 2 mM HU for 2 h or left untreated (control). After recovery for 72 hrs, the plates were incubated for 2 h in regular medium containing 40 μg/ml of neutral red and analyzed for cell viability. (**B**) Cell viability in response to H_2_O_2_. The procedures are like (**A**) except those cells were treated with 80 μM H_2_O_2_ for 1 hr. The percentages (%) of control are the ratios of colony numbers of treated cells over those of untreated cells. Cell viability was scored from three experiments. The error bars reported are the standard deviations of the averages and P-value was calculated using ANOVA followed by separate post hocs analysis. ** and *** represent *P* <0.05 and *P* <0.01, respectively.

To analyze *in vivo* functional of Rad9A DNA-binding mutants, we expressed hRad9A full-length wild-type or mutant proteins as FLAG-hRad9A in human *rad9A* KO PC3 cells. As shown previously [49, 50], the *rad9A* KO PC3 cells do not abrogate endogenous Rad9 protein completely (S1A Fig). The levels of ectopically expressed FLAG-hRad9A proteins were approximately the same but were about 30% the levels of endogenous hRad9 in *rad9A*^+/+^ PC3 cells (S1A Fig). Their cellular sensitivities to HU and H_2_O_2_ were measured. Human *rad9A* KO cells had a lower cell viability than the control *rad9A*^+/+^ PC3 cells after 2 mM HU and 80 μM H_2_O_2_ treatment and three-day recovery (Fig 5A and 5B, compare columns 1 and 2). Overexpression of WT-hRad9A^full^ rendered the *rad9A* KO cells more resistant to HU and H_2_O_2_ than KO cells transfected with vector alone (Fig 5A and 5B, compare columns 2 and 3). However, their viabilities were lower than those of *rad9A*^+/+^ PC3 cells (Fig 5A and 5B, compare columns 1 and 3). These may be caused by lower levels of ectopically expressed FLAG-hRad9A proteins as compared to that in *rad9A*^+/+^ PC3 cells (S1A Fig). When the DM-Rad9A^full^ mutant or K220A-Rad9A^full^ mutant was expressed in *rad9A* KO PC3 cells, these cells were more sensitive to H_2_O_2_, but not HU, than *rad9A* KO cells expressing wild-type hRad9A (Fig 5A and 5B, compare columns 3 and 4, compare columns 3 and 6). Lastly when the LD-Rad9A^full^ mutant was expressed in *rad9A* KO cells, it was more sensitive to both HU and H_2_O_2_ than *rad9A* KO cells expressing wild-type hRad9A (Fig 5A and 5B, compare columns 3 and 5). Thus, R150A/R223A and K220A mutations affect cellular sensitivity to only H_2_O_2_, but not HU, while loop deletion affects cellular sensitivity to both HU and H_2_O_2_. In summary, Rad9A binding to DNA is important for cellular response to oxidative stress.

### Cells with Rad9A mutation are defective in checkpoint signal activation

**Fig 6:**
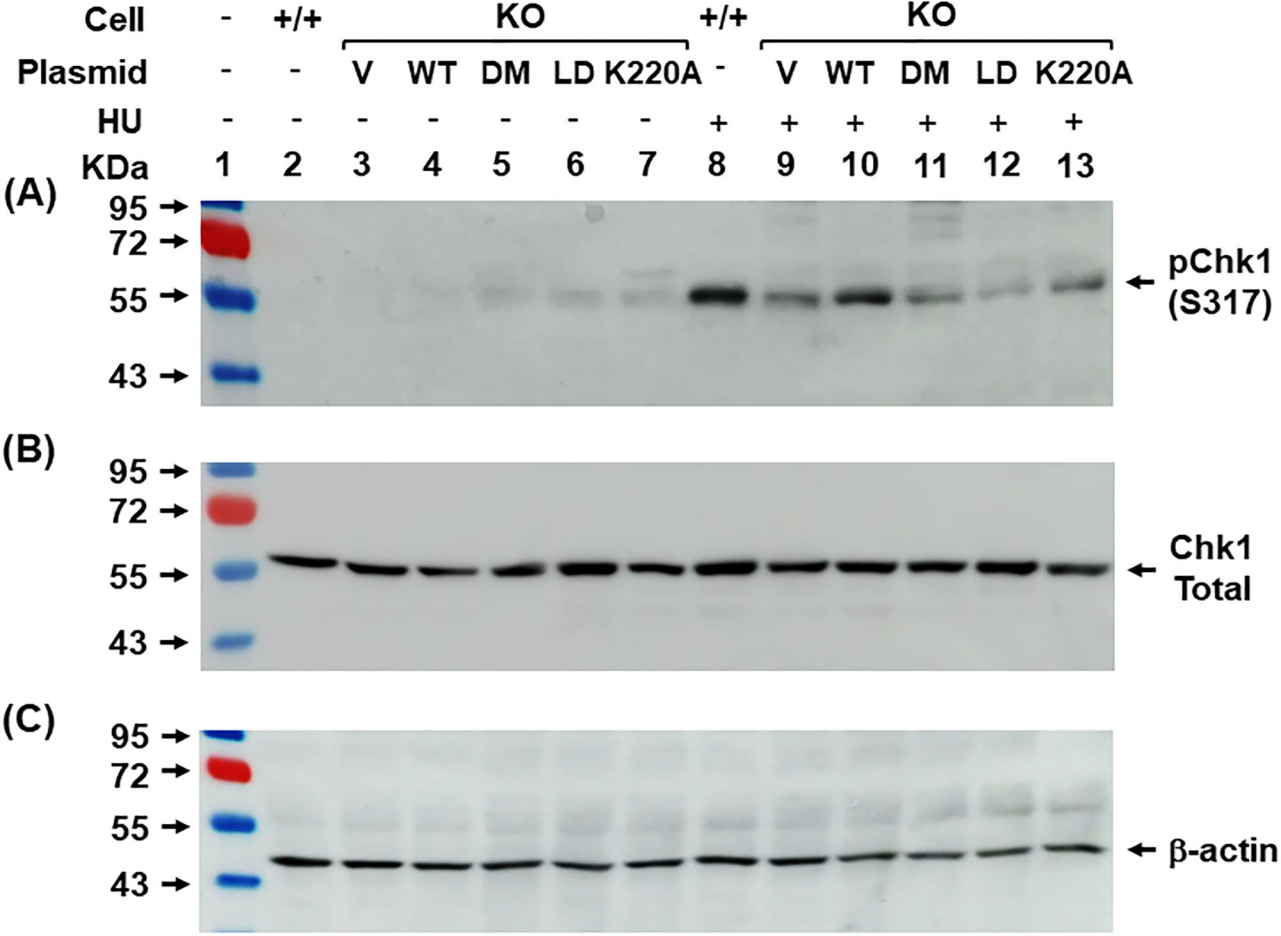
DNA association of Rad9A is required for HU-induced Chk1 phosphorylation. The control *rad9A*^+/+^ (+/+) and *rad9A*^-/-^ (KO) PC3 cells transfected with vector (V), FLAG-WT-hRad9A (WT), FLAG-DM-hRad9A (DM), FLAG-LD-hRad9A (LD) or FLAG-K220A-hRad9A (K220A) were treated with 16 mM hydroxyurea (HU) for 2 hours and recovered for 2 hours or left untreated. Cell extracts were subjected to Western blotting analysis with antibodies against phosphorylated Chk1 (pChk1) at Ser317 (S-317) (**A**), total Chk1 (**B**), and β-actin (**C**). Molecular weight markers were loaded on lane 1 with indicated sizes in KDa.

To determine whether Rad9A mutants affect checkpoint signaling, we assessed HU-induced phosphorylation of Chk1, a downstream target of ATR. Wild-type and mutant hRad9A proteins were expressed as FLAG-Rad9A^full^ in *rad9A* KO PC3 cells and the levels Chk1 phosphorylation (pChk1) at Ser317 were analyzed after treatments with 16 mM HU and 2-hour recovery. The pChk1 levels at Ser317 were very low in all HU-untreated cells, however, they were slightly higher in Rad9A-transfected *rad9A* KO cells (Fig 6A, lanes 2–7). After HU treatment, the level of Chk1 phosphorylation was highly elevated in HU-treated *rad9A*^+/+^ cells (Fig 6A, lane 8). As reported previously for *rad9* KO mouse embryonic stem cells [9], *rad9A* KO human PC3 cells were impaired for Chk1 phosphorylation at S317 upon HU treatment (Fig 6A, compare lanes 8 and 9). The residual Chk1 phosphorylation in *rad9A* KO PC3 cells may be caused by incomplete Rad9 protein depletion (S1A Fig) or 9-1-1 independent DDR. This defect of the pChk1 response in *rad9A* KO PC3 cells was partially complemented by expressing WT-hRad9A^full^ (Fig 6A, compare lanes 8–10). When DM-hRad9A^full^, LD-hRad9A^full^ and K220A-hRad9A^full^ were expressed in *rad9A* KO cells, the HU-induced pChk1 levels at S317 were reduced compared to KO cells expressing WT-Rad9A^full^ (Fig 6A, compare lane 10 to lanes 11–13). The HU-induced pChk1 levels in *rad9A* KO cells expressing DM and K220A were similar to that of *rad9A* KO cells transfected with vector alone (Fig 6A, compare lane 9 to lanes 11 and 13). However, the HU-induced pChk1 level in *rad9A* KO cells expressing LD-Rad9A^full^ was lower than that in *rad9A* KO cells transfected with vector alone (Fig 6A, compare lanes 9 and 12). Thus, checkpoint activation was completely defective in *rad9A* KO cells expressing LD-Rad9A^full^. Our results indicate that cells with defective Rad9A-DNA interactions have significantly impaired DDR signaling activation.

## Discussion

When cells are under stress or replication block, activation of the checkpoint pathway is essential to coordinate cell cycle progression with critical cellular events. The 9-1-1 complex acts as a sensor of the DNA damage response and enhances DNA repair, and its expression is associated with cancer development and treatment [54–56]. Understanding the DNA binding properties of 9-1-1 is important for elucidating its role the checkpoint activation process. In this study, we show that both N- and C-terminal globular domains of Rad9A are involved in DNA association. Deleting any region of hRad9A has detrimental effects on its DNA binding ability as the DNA binding affinities of each half of Rad9A core are substantially weaker than that of intact Rad9A^1-266^ core. This contrasts with Hus1 DNA binding that only requires N-terminal globular domains [43] and is consistent with higher DNA affinity of hRad9A^1-266^ than Hus1 and Rad1 [43]. Rad9A interacts with DNA through positively charged residues, non-positively charged residues, and a small loop in synergistic actions. We also show that DNA association by Rad9A is critical to maintaining cell viability and checkpoint activation under oxidative stress.

We examined the DNA binding activities of Rad9A subunit in the absence of the other two subunits. It is possible that the DNA binding characteristics revealed with isolated Rad9A subunit may not reflect the binding properties of the intact 9-1-1 clamp. Nevertheless, our results provide meaningful knowledge to the roles of Rad9A on the DNA damage response and gene regulation. First, Rad9A^1-266^ can bind blunt-ended DNA with high affinity, close to that displayed by the 9^1−266^-1-1 complex [43]. The difference between Rad9A^1-266^ subunit and the 9^1−266^-1-1 complex is the preferential recruitment of 9^1−266^-1-1 to 5’-recessed DNA substrates [43]. Second, the *in vitro* properties of Rad9A mutants are supported by their defective *in vivo* complementation activities. *Rad9A* KO cells expressing DM, LD, and K220A mutants are more sensitive to hydrogen peroxide and are defective in checkpoint signal activation. Third, the DNA binding of Rad9A may be important for 9-1-1 loading process. After 9-1-1 ring is opened by Rad17-RFC_2-5_ [5–7] at the junction between Rad1 and Hus1 [35–37], strong DNA affinity of Rad9A subunit can facilitate the complex association with DNA. Finally, DNA binding of Rad9A subunit in the absence of the other two subunits may contribute to its independent role in gene regulation [21–23].

We identify basic residues K11, K15, R22, K78, K220, and R223 located on the inner ring surface of both globular domains of hRad9A as being involved in DNA binding. These basic residues are proposed to form polar interactions with DNA phosphodiester backbone. Because R150A mutant protein has poor solubility, the role of R150 in DNA association remains to be tested. Strikingly, DNA binding is synergistically reduced with multiple Ala substituted mutations of these residues and is dependent on their residential Rad9A deletion constructs. For example, R150A/R223A Rad9A^94-266^ double mutant has 6.7-fold weaker DNA binding than R223A single mutants (Table 1). Rad9A^1-266^ quadruple mutant [K11A/K15A/R22A/K78A (QM1)] is 14.8-fold weaker DNA binding than K11A/K15A/R22A Rad9A^1-266^ triple mutant (TM) (Table 1). In addition, TM-Rad9A^1-133^ has 64.2-fold weaker DNA binding than TM-Rad9A^1-266^ while WT-Rad9A^1-133^ has 16.4-fold weaker DNA binding than WT-Rad9A^1-266^ (Table 1). These properties are comparable to those found with mHus1 mutations [48]. Lim *et al*. have shown that chromatin association of mHus1 requires synergistical actions of several important basic residues [48]. They showed that Hus1^K93A^ mutant has a phenotype like WT-mHus1 and K25A/K236A/K237A mHus1 triple mutant has mild defect, while K25A/K93A/K236A/K237A Hus1 quadruple mutant completely fails to complement the sensitivity to genotoxin resistance in *Hus1* KO cells.

In addition to polar interactions, DNA binding of Rad9A may involve non-polar interactions. For example, Rad9A^94-266^ binds DNA 2-fold better than Rad9A^134-266^, but K96, R107, R119, and K120 resided within residues 94–133 are not located on the inner ring surface of the 9-1-1 complex.

Interestingly, a loop-deletion (LD) mutant (with residues 160–163 deleted) of Rad9A^94-266^ has much weaker DNA binding activity than WT-Rad9A^4-266^. The published 9-1-1 structures indicate that this small loop with sequence SPAL projects into the ring channel [35–37] (Fig 1). Because this small loop is not present in PCNA, its role in DNA binding is unique to the 9-1-1 complex. We propose that this loop may clasp the DNA in the complex either through hydrogen bonding between S160 and DNA phosphate backbone or hydrophobic interaction through L163. He *et al*. have reported that S160A mutation of hRad9A significantly reduces mismatch repair activity but has no impact on checkpoint control and cellular sensitivity to genotoxic agents [57]. Their results suggest that S160 of hRad9A may play minimal role in DNA binding. Because removing this loop may affects the overall fold of Rad9A, mutating individual residues in the loop (such as L163) is needed to clarify its function. We show that *rad9A* KO human cells expressing LD-Rad9A mutant are more sensitive to HU and H_2_O_2_ and contain lower levels of HU-induced Chk1 phosphorylation than *rad9A* KO cells transfected with vector alone. Thus, this small loop of Rad9A plays an important role in 9-1-1 loading onto DNA, DNA repair, and checkpoint activation. How LD-Rad9A mutation causes the Chk1 signaling defect requires further investigation.

**Fig 7:**
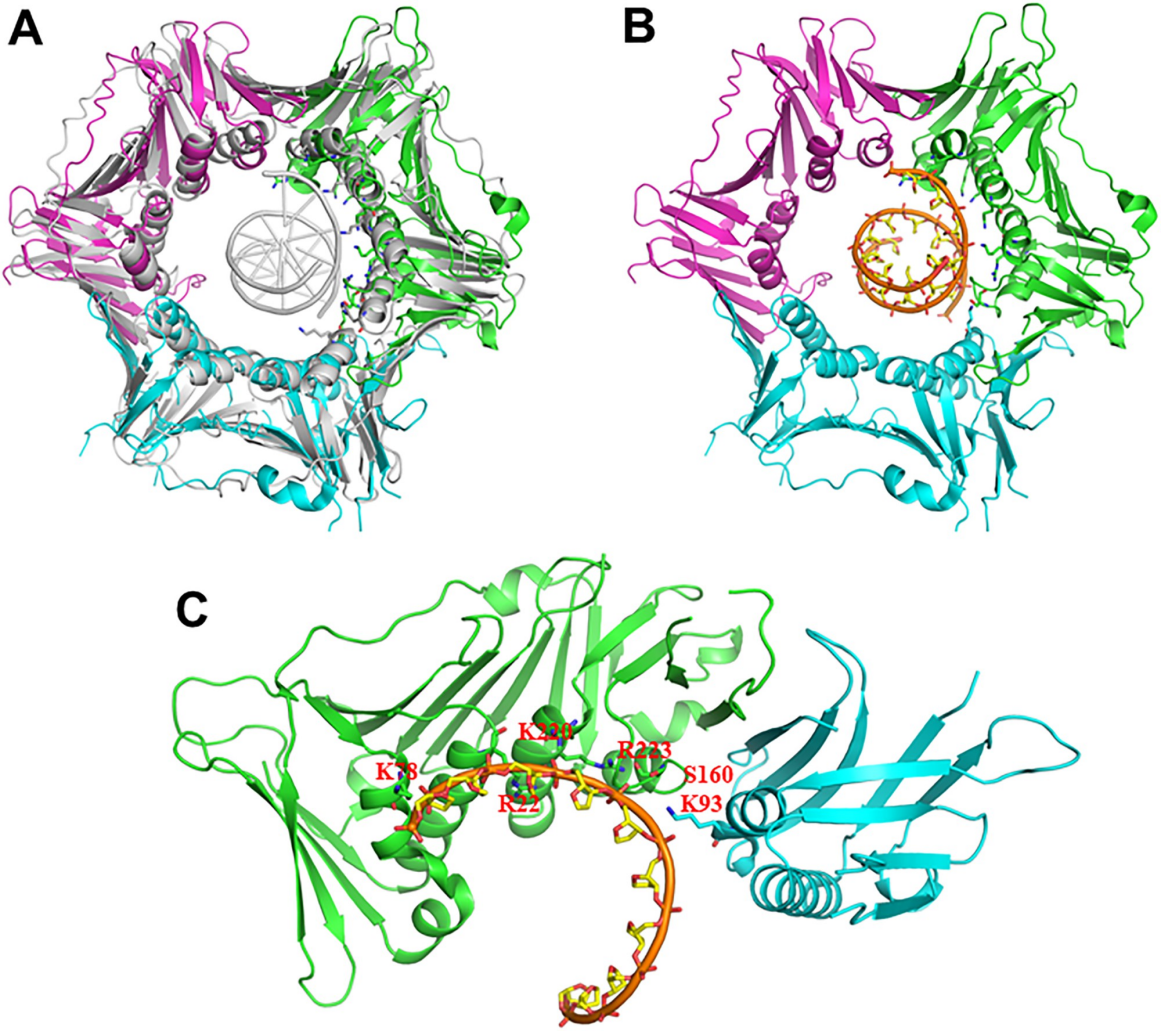
A model of the 9-1-1 complex with DNA based on PCNA-DNA structure. (**A)** Human 9-1-1 (access # 3A1J) [37] is superimposed to PCNA-DNA structure (access # 5L7C) [47]. Rad9A, Hus1, Rad1, and PCNA are colored in green, blue, magenta, and gray, respectively. DNA is shown in gray. (**B**) PCNA protein structure is omitted from (**A**). DNA is shown in orange. (**C**) Superimposed structure of Rad9A, N-terminal domain of Hus1, and DNA. Side chains of R22, K78, S160, K220, and R223 of hRad9A and K93 of hHus1 are shown.

Structural comparison and evolutionary conservation analysis suggest that Rad9A of 9-1-1 is the most closely related subunit to PCNA [36, 48]. De March *et al*. recently showed that PCNA-DNA complex interface involves the side chains of five basic residues (K20, K77, R149, H153, and K217), distributed on four α-helices of one hPCNA subunit and another residue (K80) on the proximal α-helix of the adjacent subunit [47]. These authors also showed that the DNA contacted residues are arranged in a right-hand spiral that matches the pitch of B-DNA and proposed that PCNA clamp slides by tracking the DNA backbone via a “cogwheel” mechanism [47]. Based on their results, we model the 9-1-1/DNA binding with four α-helices of Rad9A and adjacent helix H2 of Hus1 (Fig 7). In this model, K78, R22, K220, and R223 of hRad9A, and K93 of hHus1 are arranged in a right-hand spiral that matches the pitch of B-DNA (Fig 7C). We predict that R85 and R150 may play minimal roles in DNA association because they are not located on the spiral path. Remarkably, the small loop (residues 160–163) of hRad9A is positioned on the path of this spiral.

However, this PCNA-DNA based modeling of the 911 complex on DNA does not agree with the modeling of Querol-Audi *et al*. who have used an integrative computational and experimental approach to define the assemblies of FEN1 with either PCNA or 9-1-1 on double-flap DNA substrates [45]. Their results showed that 9-1-1 displays tight association with DNA, forming 13 stable contacts with the DNA backbone (compared with nine contacts in the PCNA complex). Unlike PCNA, all three subunits of the 9-1-1 complex contribute to binding both DNA strands. Their model shows that three basic residues (R85, R22, and R223) of Rad9A contact one DNA strand and K92 of Rad9A contacts the other DNA strand [45]. Their proposed model is substantially different from that derived from our results. These differences may stem from the use of different DNA substrates (double-flap DNA vs. blunt-ended DNA), protein components (9-1-1 ring vs. individual subunits), and the presence or absence of FEN1 binding partner. It has been shown that 9-1-1 has a distinctive preference for 5’-recessed DNA [5–7, 43] while Rad9A and Hus1 have no such preference [43]. Therefore, further structural analyses of 9-1-1/DNA complex is needed.

We demonstrate that defective DNA binding of Rad9A is accompanied by sensitivity to H_2_O_2_ as well as reduced HU-induced Chk1 phosphorylation. We show, for the first time, that *rad9A* KO human cells have a lower viability than the control *rad9A*^+/+^ cells after H_2_O_2_ treatment. Cellular sensitivity to oxidative stress of *rad9A* KO cells is only partially restored by expressing WT-Rad9A^full^ because the level of ectopically expressed FLAG-hRad9A protein is lower than endogenous Rad9 in *rad9A*^+/+^ PC3 cells (S1A Fig). Three mutants (K220A, DM, and LD) we tested are unable to compensate the H_2_O_2_ sensitivity phenotype of *rad9A* KO cells.

As for HU sensitivity, *rad9A* KO cells expressing K220A and DM behave like cells expressing wild-type Rad9A while cells expressing the LD mutant are sensitive to HU. The pChk1 response in LD mutant expressing cells is also different from those of K222A and DM mutants. The HU-induced pChk1 levels at S317 in *rad9A* KO cells expressing LD-Rad9A^full^ is lower than those in cells expressing K222A and DM mutants which are similar to those in cells transfected with vector alone (Fig 6A). Since the DNA binding affinities of K220A, DM, and LD Rad9 mutants are reduced to a comparable extent, their different sensitivities to HU may be explained by the level of P-Chk1 level in *rad9A* KO cells expressing LD mutant being lower than those of cells expressing K220A and DM mutants under HU treatment. It is notable that K220A and DM mutants have significant pChk1 defects but are not hypersensitive to HU. These may be caused by the differences in HU dose used for the two assays. The experiments of HU sensitivity were performed with 2 mM HU treatment and 3-day recovery while the experiments of pChk1 activation were performed with 16 mM HU and two-hour recovery. The differences in pChk1 activation only become evident at high HU doses that are greater than those used in the survival assays.

Our findings are consistent with the report that loss of DNA contacts for mHus1 leads to severe checkpoint signaling defects and hypersensitivity to genotoxins [48]. Lim *et al*. have shown that reduction of mHus1 function requires mutations of more than four important basic residues [48]. Remarkably, our results indicate that single and double mutations of basic residues and deletion of a small loop of hRad9A have significant reduction on DNA binding and can lead to loss of complementation to genotoxin sensitivity in *rad9A* KO cells. This may be explained by Rad9A’s higher DNA affinity over the Hus1 subunit [43]. Therefore, both Rad9A-DNA and Hus1-DNA interactions are required for proper loading of 9-1-1 on damaged DNA and Rad9A plays more profound role in DNA association and DDR signal transduction.

## Supporting information

S1 Fig Expression of Rad9 protein in *rad9* KO cell. Cell extracts were prepared from the control *rad9A*^+/+^ (+/+) and *rad9A*^-/-^ (KO) PC3 cells transfected with vector (V), FLAG-WT-hRad9A (WT), FLAG-DM-hRad9A (DM), FLAG-LD-hRad9A (LD) or FLAG-K220A-hRad9A (K220A) and subjected to Western blotting analysis to detect total Rad9 (**A**) and β-actin (**B**). Total Rad9A was detected by unphosphorylated monoclonal antibody #14484 (Rad9A (D2J4P) from Cell signaling Technology. Lanes 2 and 3 of (**A**) show endogenous Rad9A protein in *rad9A*^+/+^ cells and *rad9A*^-/-^ cells transfected with vector alone, respectively. The *rad9A* KO PC3 cells do not abrogate endogenous Rad9 protein completely. A non-specific band was observed below the Rad9A band. Molecular weight markers were loaded on lane 1 with indicated sizes in KDa.
(TIF)

S1 Table Oligonucleotides used. (PDF)

S1 Raw images (PDF)
