# Supplementary figures and images for: DNA binding by the Rad9A subunit of the Rad9-Rad1-Hus1 complex
Source: PLoS One. 2022 Aug 8;17(8):e0272645. doi: 10.1371/journal.pone.0272645 (PMC9359528; doi:10.1371/journal.pone.0272645)

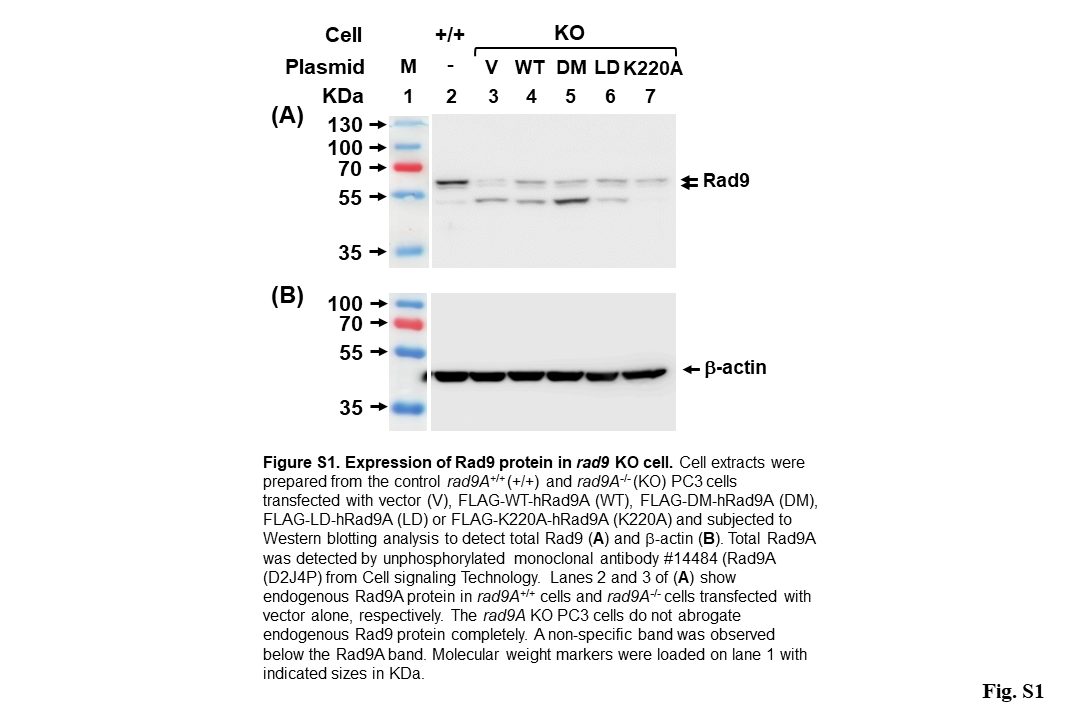

Supplement: S1 Fig — Cell extracts were prepared from the control rad9A+/+ (+/+) and rad9A-/- (KO) PC3 cells transfected with vector (V), FLAG-WT-hRad9A (WT), FLAG-DM-hRad9A (DM), FLAG-LD-hRad9A (LD) or FLAG-K220A-hRad9A (K220A) and subjected to Western blotting analysis to detect total Rad9 (A) and β-actin (B). Total Rad9A was detected by unphosphorylated monoclonal antibody #14484 (Rad9A (D2J4P) from Cell signaling Technology. Lanes 2 and 3 of (A) show endogenous Rad9A protein in rad9A+/+ cells and rad9A-/- cells transfected with vector alone, respectively. The rad9A KO PC3 cells do not abrogate endogenous Rad9 protein completely. A non-specific band was observed below the Rad9A band. Molecular weight markers were loaded on lane 1 with indicated sizes in KDa. (TIF) [file pone.0272645.s001.tif]
