# Supplementary material for: DNA binding by the Rad9A subunit of the Rad9-Rad1-Hus1 complex
Source: PLoS One. 2022 Aug 8;17(8):e0272645. doi: 10.1371/journal.pone.0272645 (PMC9359528; doi:10.1371/journal.pone.0272645)
Supplement: S1 Table — (PDF) [file pone.0272645.s002.pdf]

SUPPLEMETARY ONLINE DATA

Table S1. Oligonucleotides used

Name	Sequence	Purpose
HC40	Fl-ccgaggaattcgccttctgctccatagccactggttgtag	DNA binding substrate
HG40	ctacaaccagtggctatggagcagaaggcgaattcctcgg	DNA binding substrate
Rad9A$^{1-266}$-F	ccaggatccgatgaagtgcctggtcacgggcgg	Rad9$^{1-266}$ 5' primer
Rad9A^{1-266-}R	gtggtcgactcagtctgagagtgtggccaagac	Rad9$^{1-266}$ 3' primer
Rad9A$^{134-266}$-F	ccaggatccgatggccgtcttcgacccagcctc	Rad9$^{134-266}$ 5' primer
Rad9A^{1-133-}R	gtggtcgactcactgcagggactcacagtcctg	Rad9$^{1-133}$ 3' primer
Rad9A K11/K15A-F	ggcggcaacgtggcggtgctcggcgcggccgtccactc	Mutagenesis primer
Rad9A K11/K15A-R	gagtggacggccgcgccgagcaccgccacgttgccgcc	Mutagenesis primer
Rad9A R22A-F	ccactccctgtccgccatcggggacgag	Mutagenesis primer
Rad9A R22A-R	ctcgtccccgatggcggacagggagtgg	Mutagenesis primer
Rad9A K78A-F	gcgctgtaagatcctgatggcgtctttcctgtctgtcttc	Mutagenesis primer
Rad9A K78A-R	gaagacagacaggaaagacgccatcaggatcttacagcgc	Mutagenesis primer
Rad9A R150A-F	ccgcgccccagccgcggttctgggg	Mutagenesis primer
Rad9A R150A-R	ccccagaaccgcggctggggcgcgg	Mutagenesis primer
Rad9A ∆160-163-F	cagcgtcacttcagcgaagggcagaacagc	Mutagenesis primer
Rad9A ∆160-163-R	gctgttctgcccttcgctgaagtgacgctg	Mutagenesis primer
Rad9A K220A-F	gagcccccggaattccgcgaggcagaaagtgatg	Mutagenesis primer
Rad9A K220A-R	catcactttctgcctcgcggaattccgggggctc	Mutagenesis primer
Rad9A R223A-F	tctgcctcaaggaattcgcggggctcctgag	Mutagenesis primer
Rad9A R223A-R	ctcaggagccccgcgaattccttgaggcaga	Mutagenesis primer
Rad9A 11-223-F	gcctggtcacgggcggcaacgt	Ligation free primer
Rad9A 11-223-R	caagtttgctgactctgcaaagctc	Ligation free primer
Linearize-F	gagtcagcaaacttgaatcttagcatt	Ligation free primer
Linearize-R	cgcccgtgaccaggcacttca	Ligation free primer

[a] Fl indicates the DNA 5′-end labeled with a fluorescein (6-carboxyfluorescein).
