# Supplementary material for: DNA binding by the Rad9A subunit of the Rad9-Rad1-Hus1 complex
Source: PLoS One. 2022 Aug 8;17(8):e0272645. doi: 10.1371/journal.pone.0272645 (PMC9359528; doi:10.1371/journal.pone.0272645)

# S1 Raw Images

- **Lanes marked with X not included in final figure**
- **Lanes marked with a triangle in the final figure**
- **Pages 2-11: Figure 3 Raw Images**
- **Pages 12-14: Figure 6 Raw Images**
- **Page 15: Supplemental figure 1 Raw Images**

**Figure 3 WT Rad9A 1-266: Fractions from CHT Column. Fraction 48 was used as the representative lane for this protein construct**

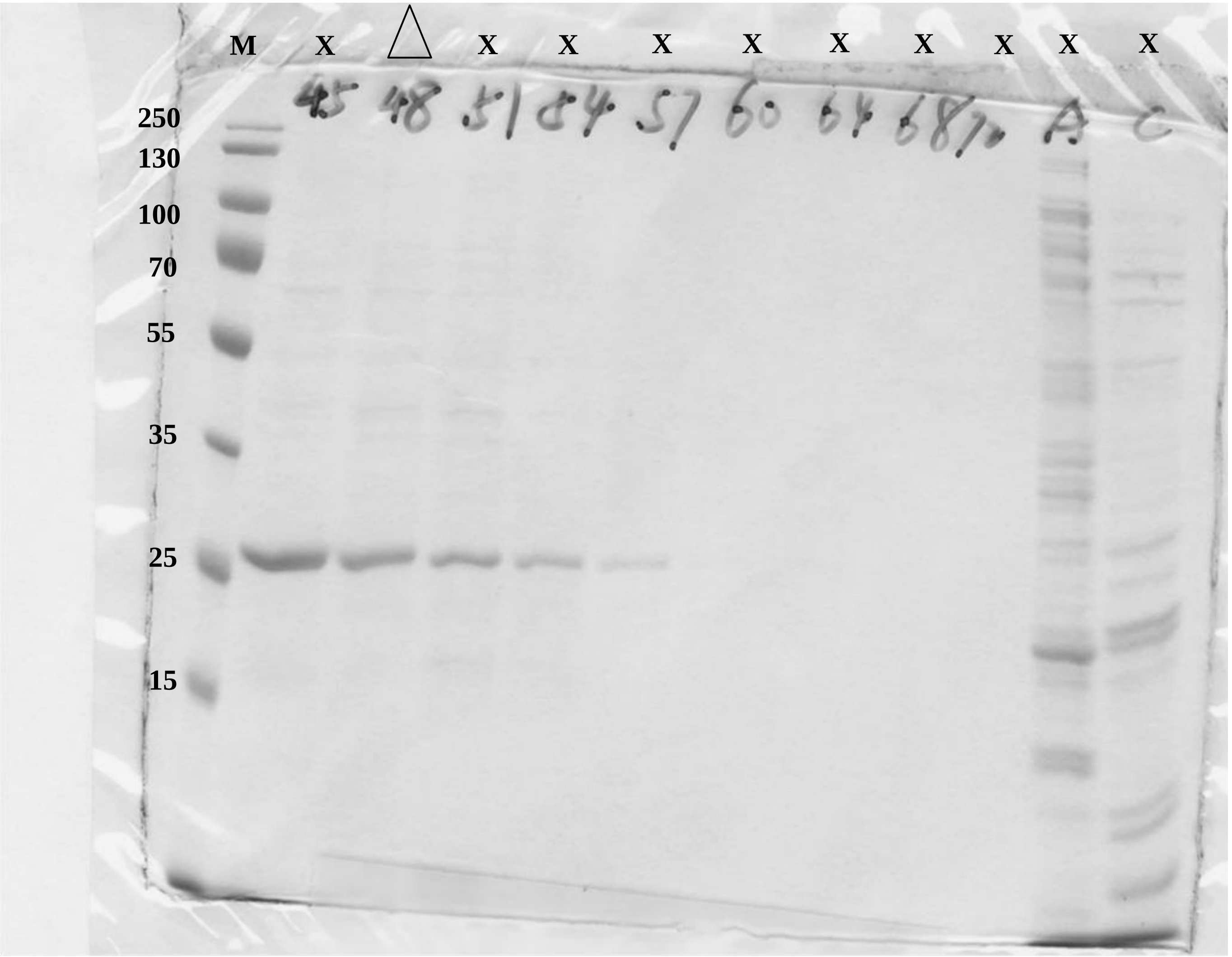

**Figure 3 WT Rad9A$^{1\text{-}133}$ : BSA concentration gradient followed by varying concentrations of WT Rad9A$^{1\text{-}133}$**

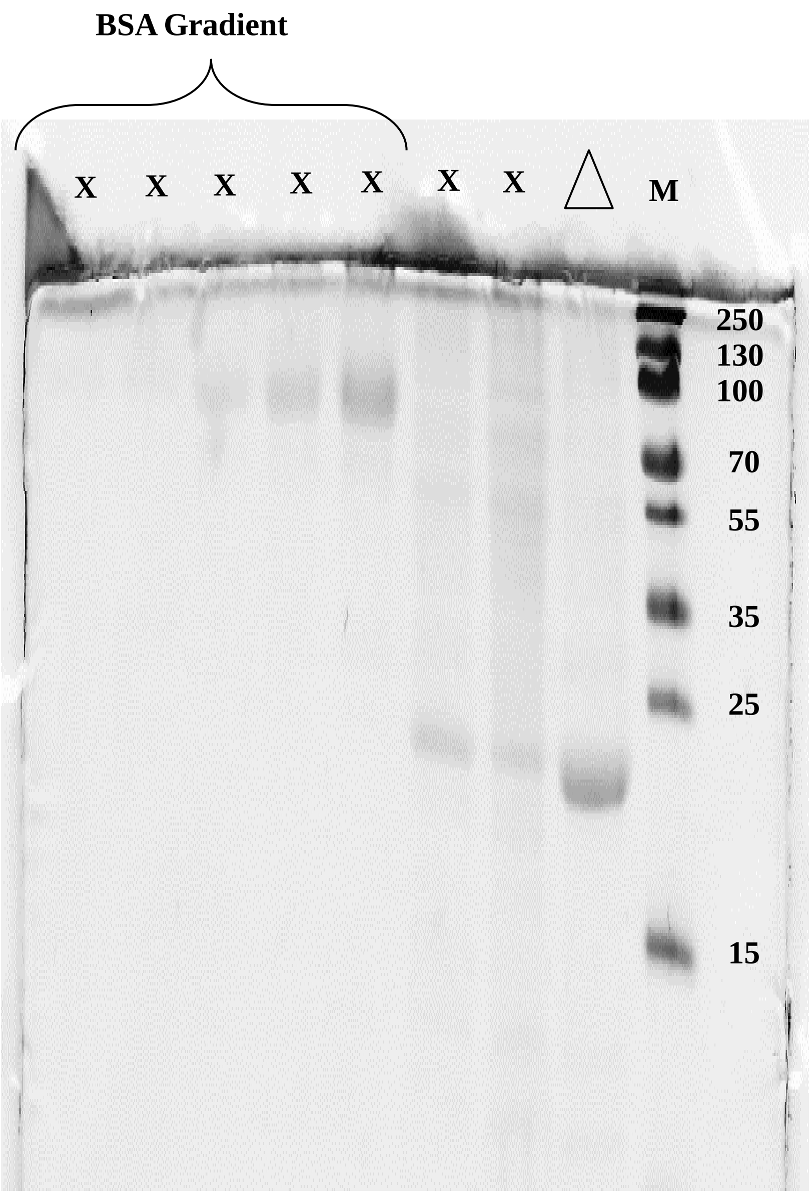

**Figure 3 WT 134-266: Varying concentrations of RAD9A WT 134-266 followed by a BSA gradient.**

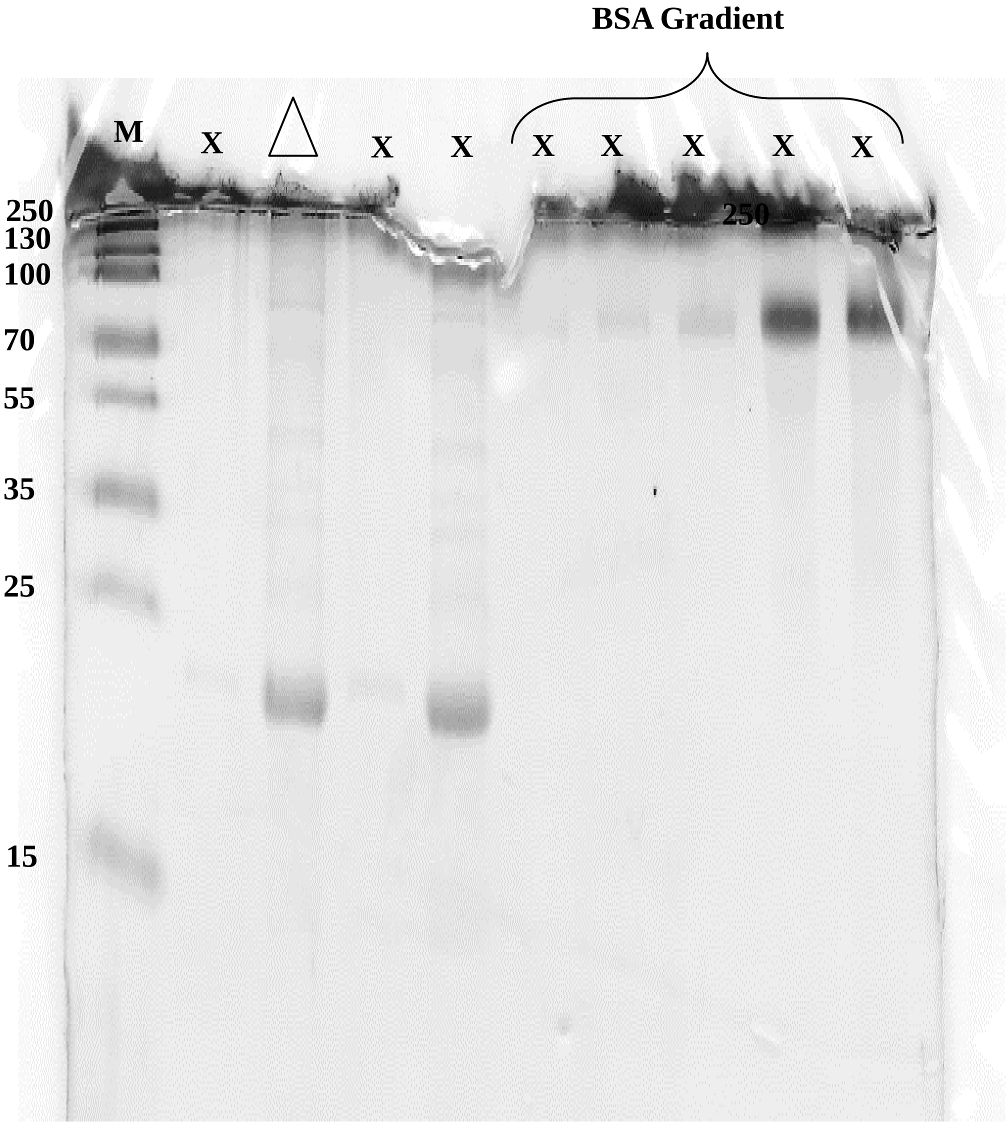

**Figure 3 WT, DM and LD Rad9A$^{94-266}$**

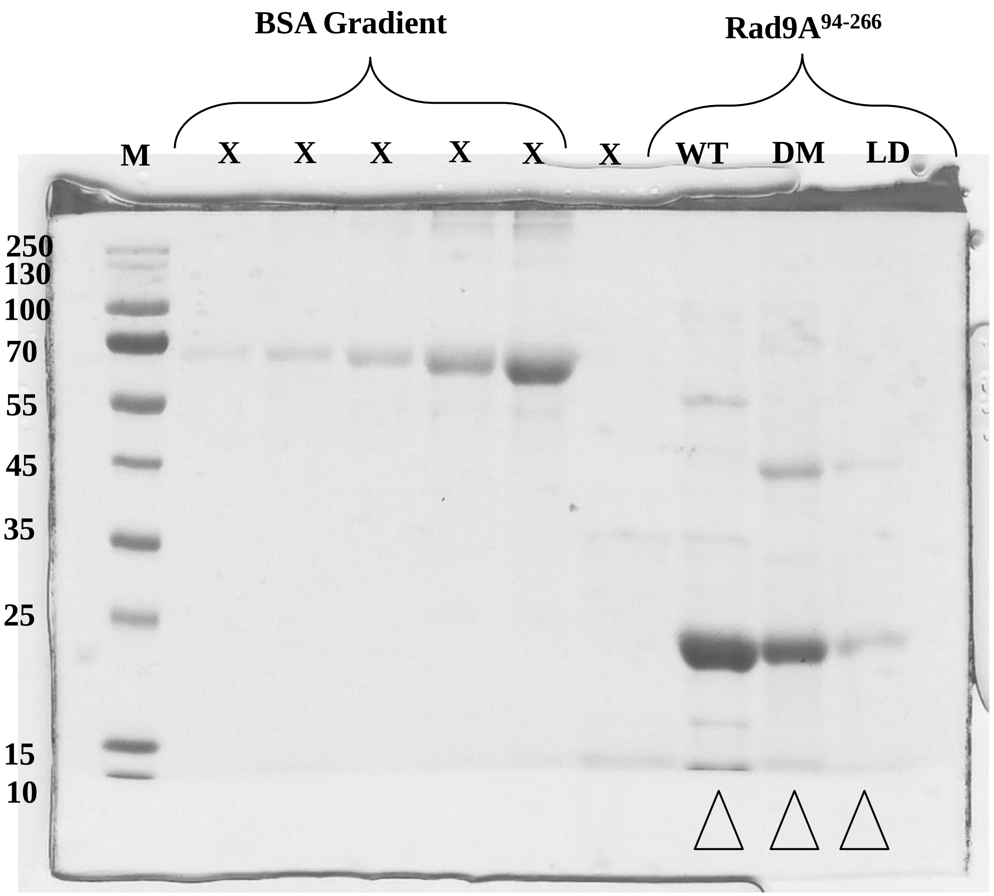

**Figure 3 K220A Rad9A$^{94-266}$**

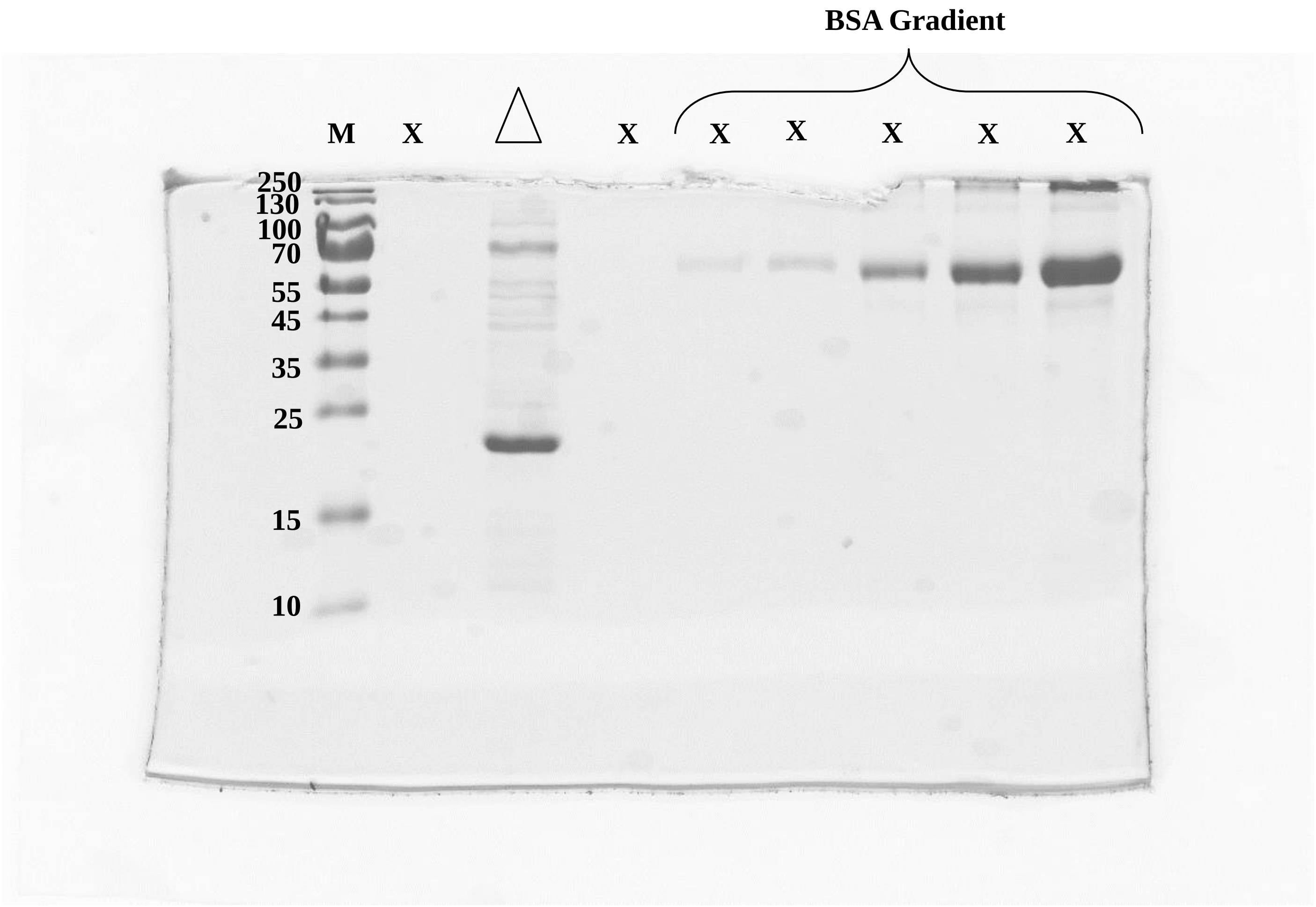

**Figure 3 R150A Rad9A$^{94-266}$**

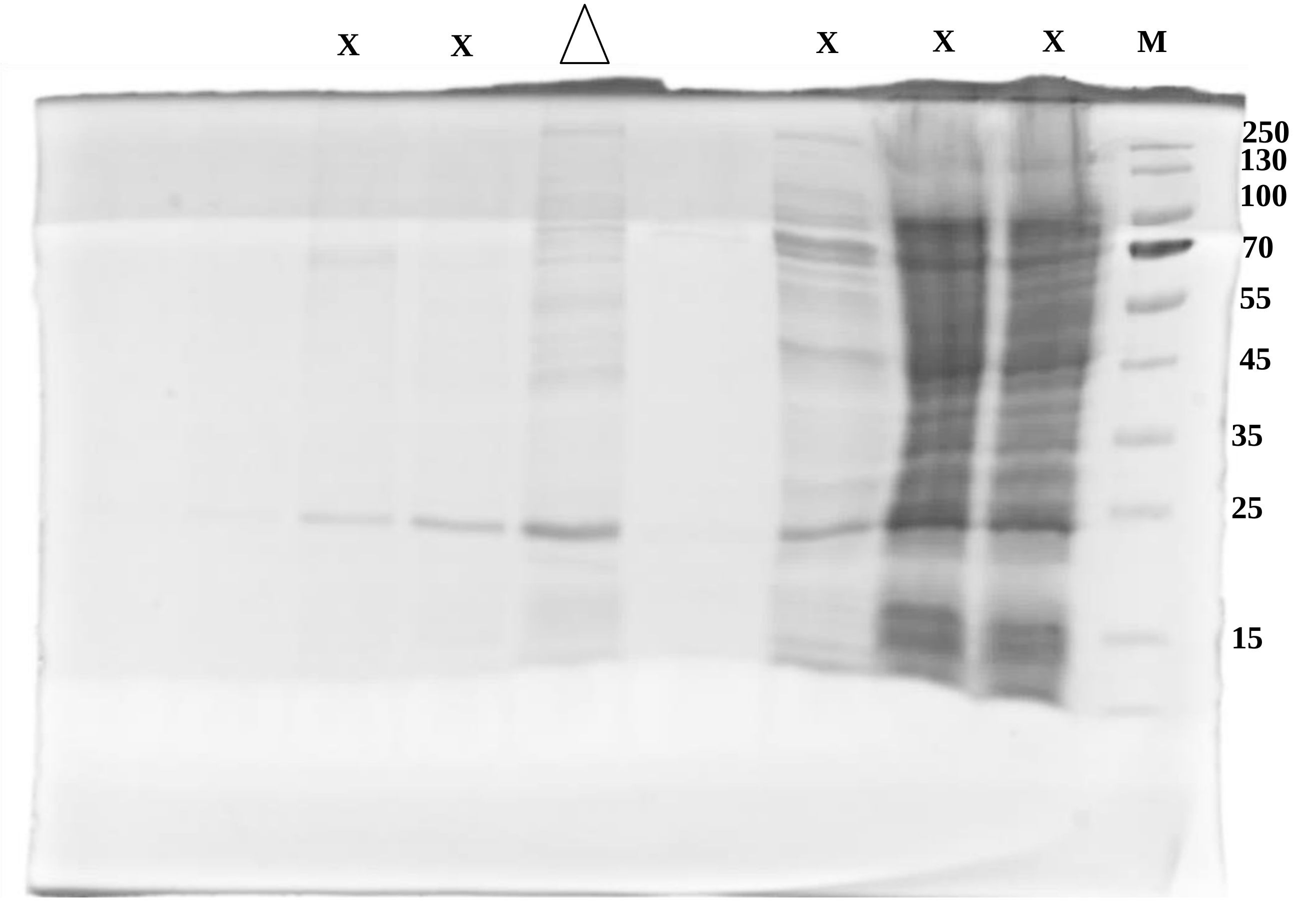

**Figure 3 R223A Rad9A$^{94\text{-}266}$**

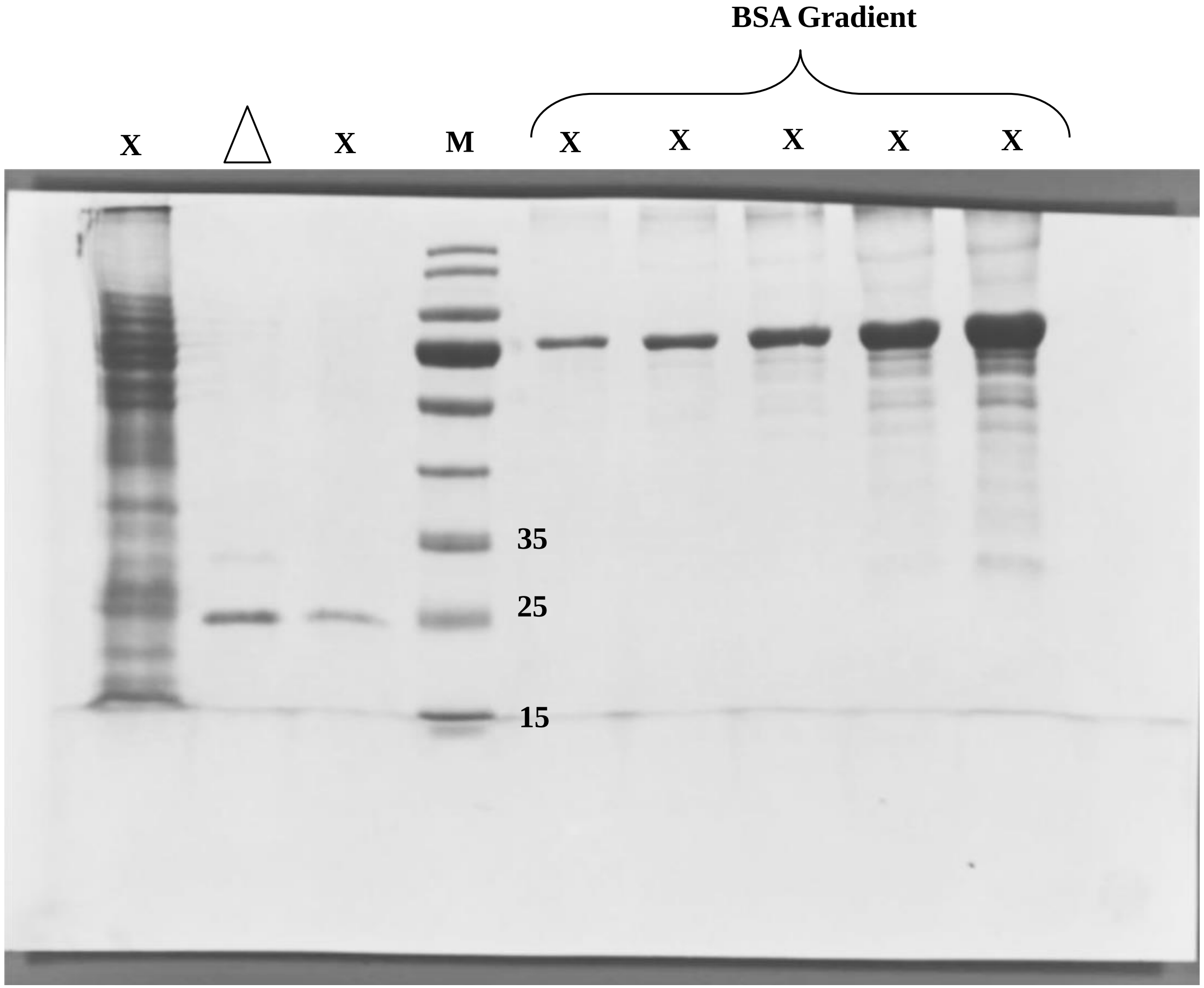

Figure 3 TM Rad9A$^{1-266}$

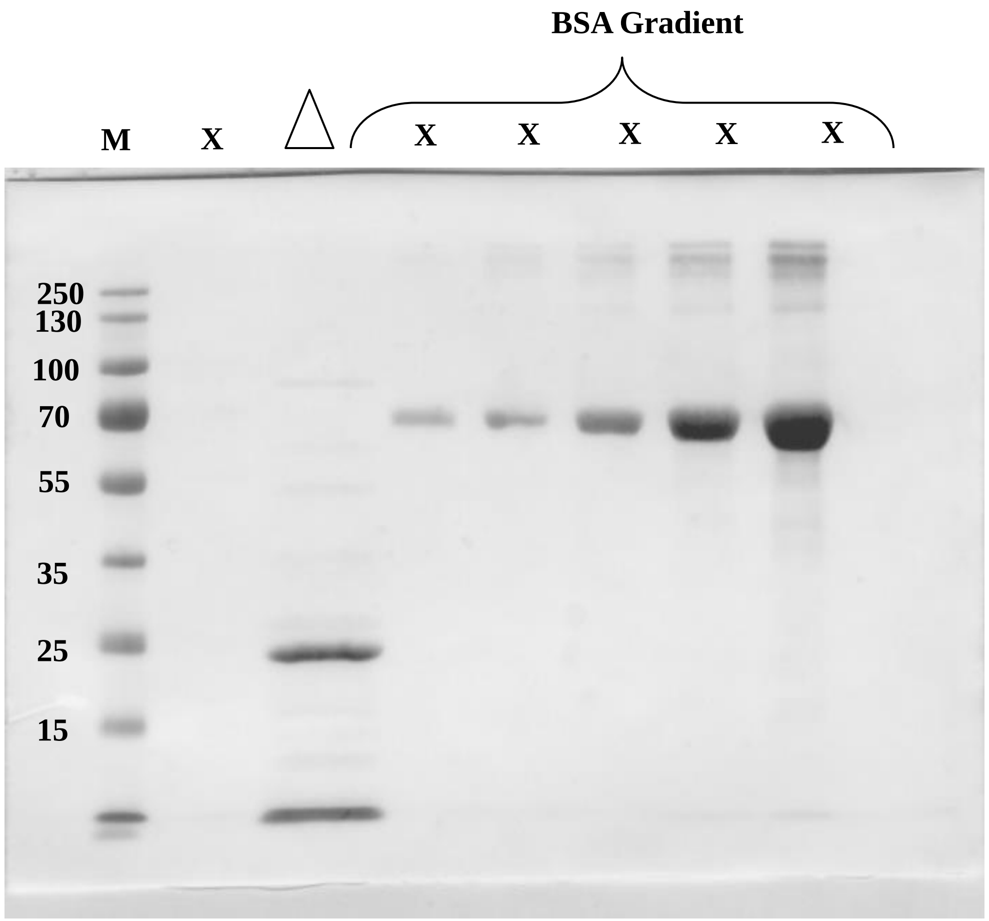

**Figure 3 TM Rad9A$^{1-133}$ Fractions from a CHT column**

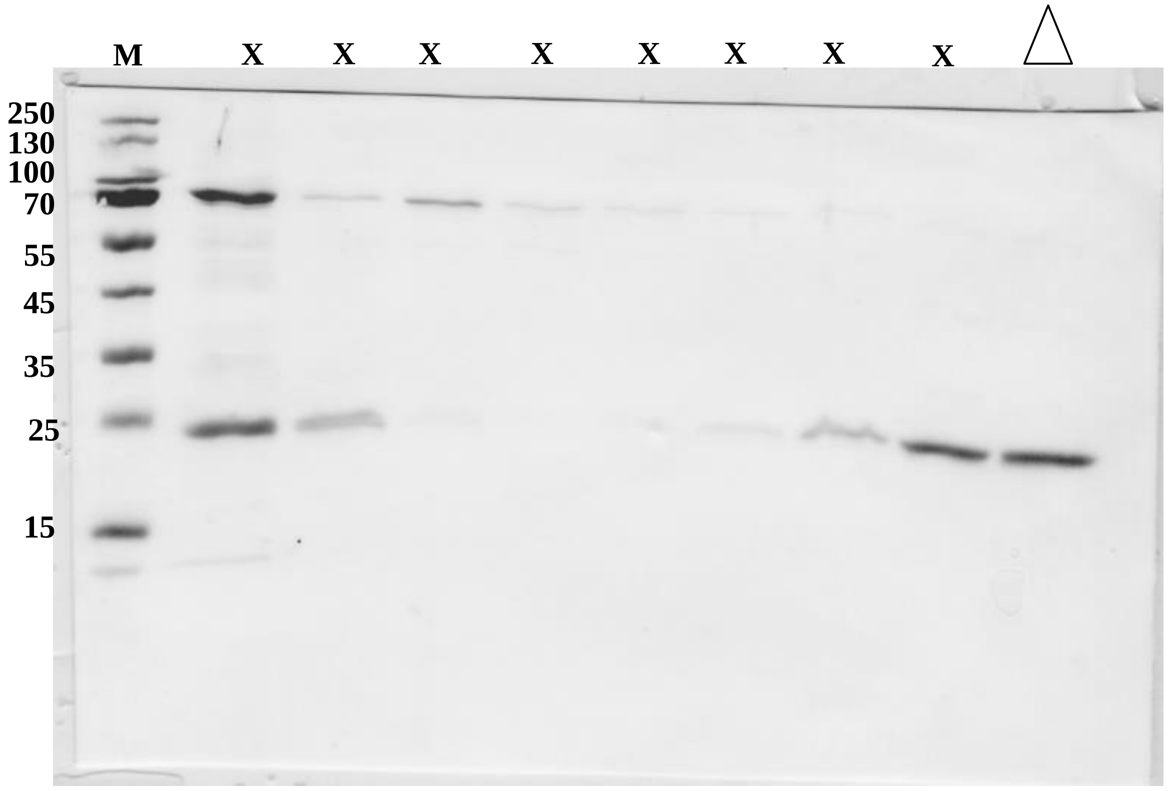

**Figure 3 QM1 Rad9A$^{1-266}$ Commassie Stain of fractions from CHT column and IMAC column**

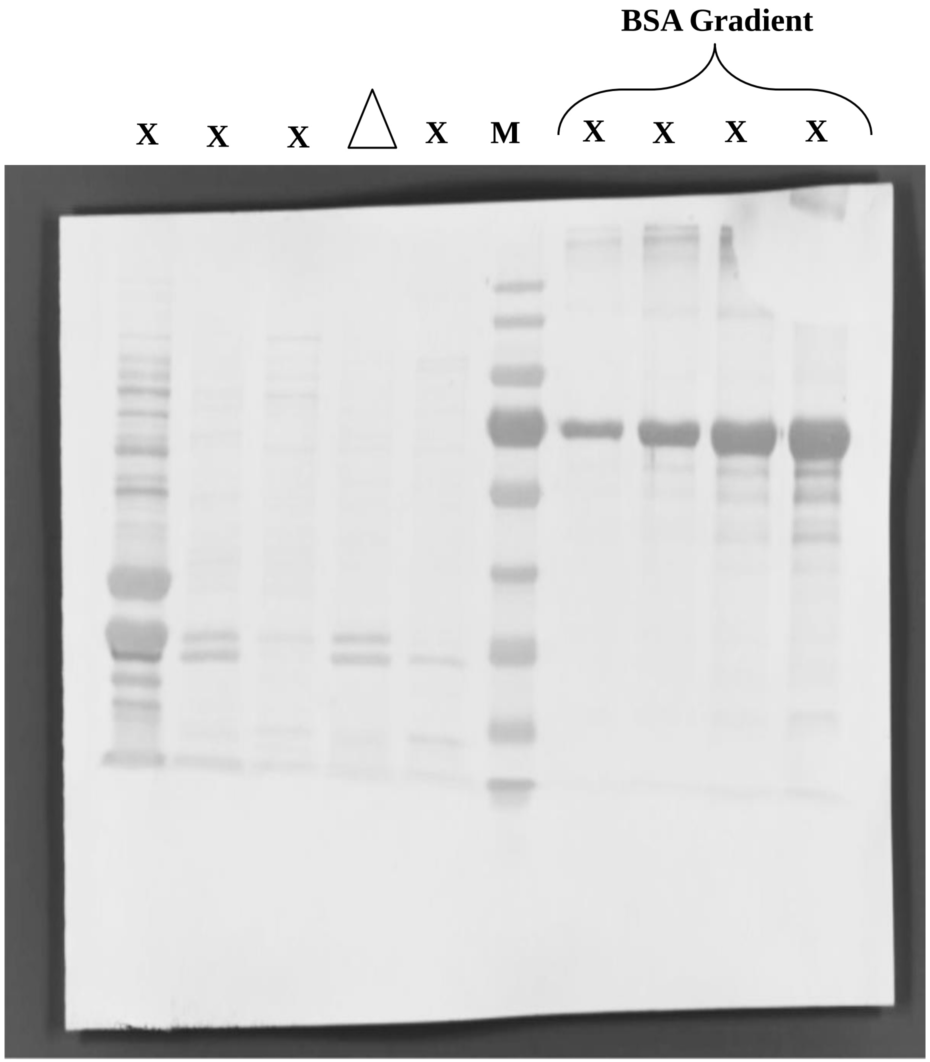

**Figure 6a: pCHk1 (S317) immunoblot. All Lanes were used in the making of this figure. Treatment with 16 mM HU for 2 hours followed by 2 hours recovery compared to no HU treatment.**

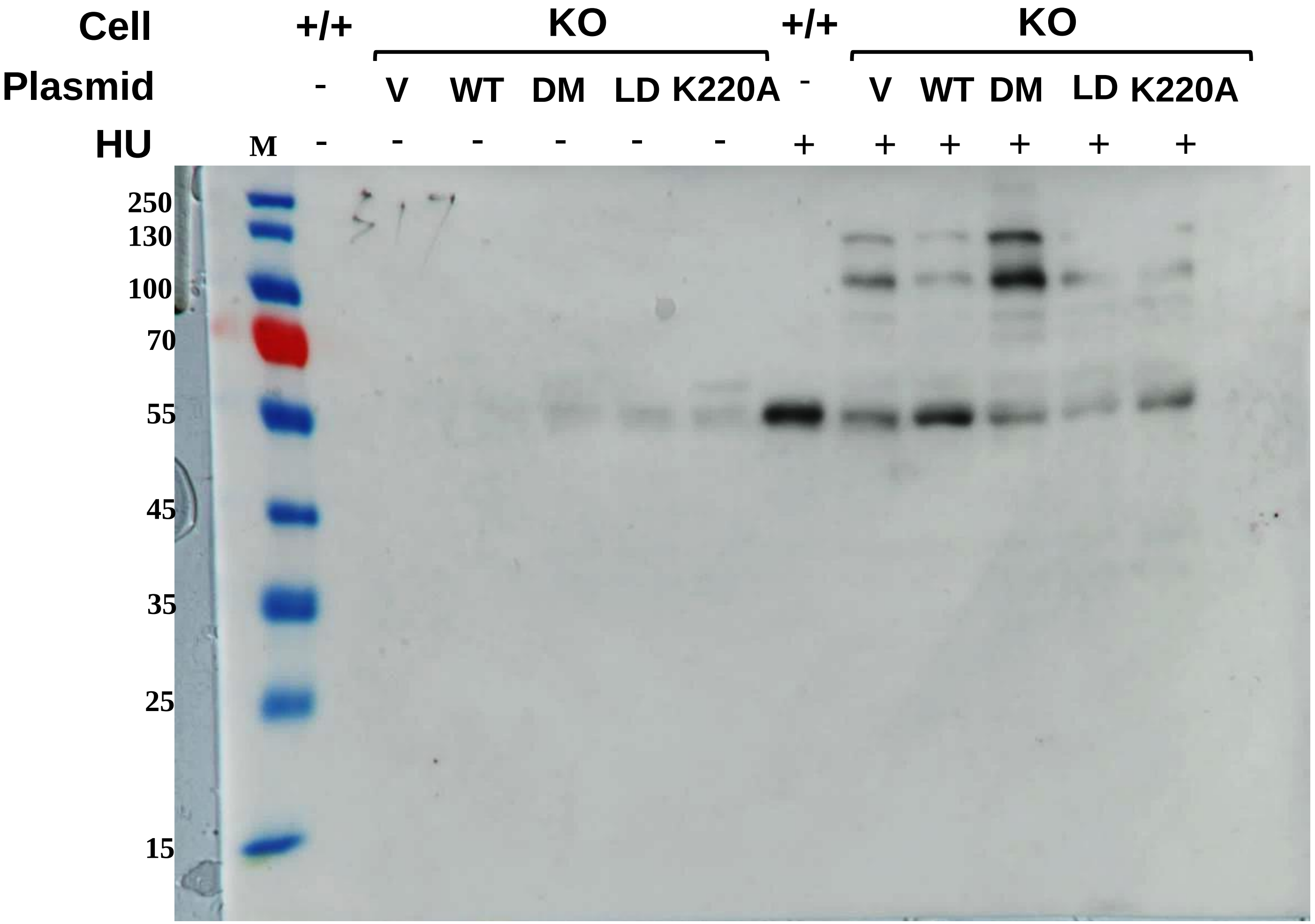

**Fig 6b Immunoblot of total Chk1 in the varying cell lines. All lanes were used in this figure. Treatment with 16 mM HU for 2 hours followed by 2 hours recovery compared to no HU treatment.**

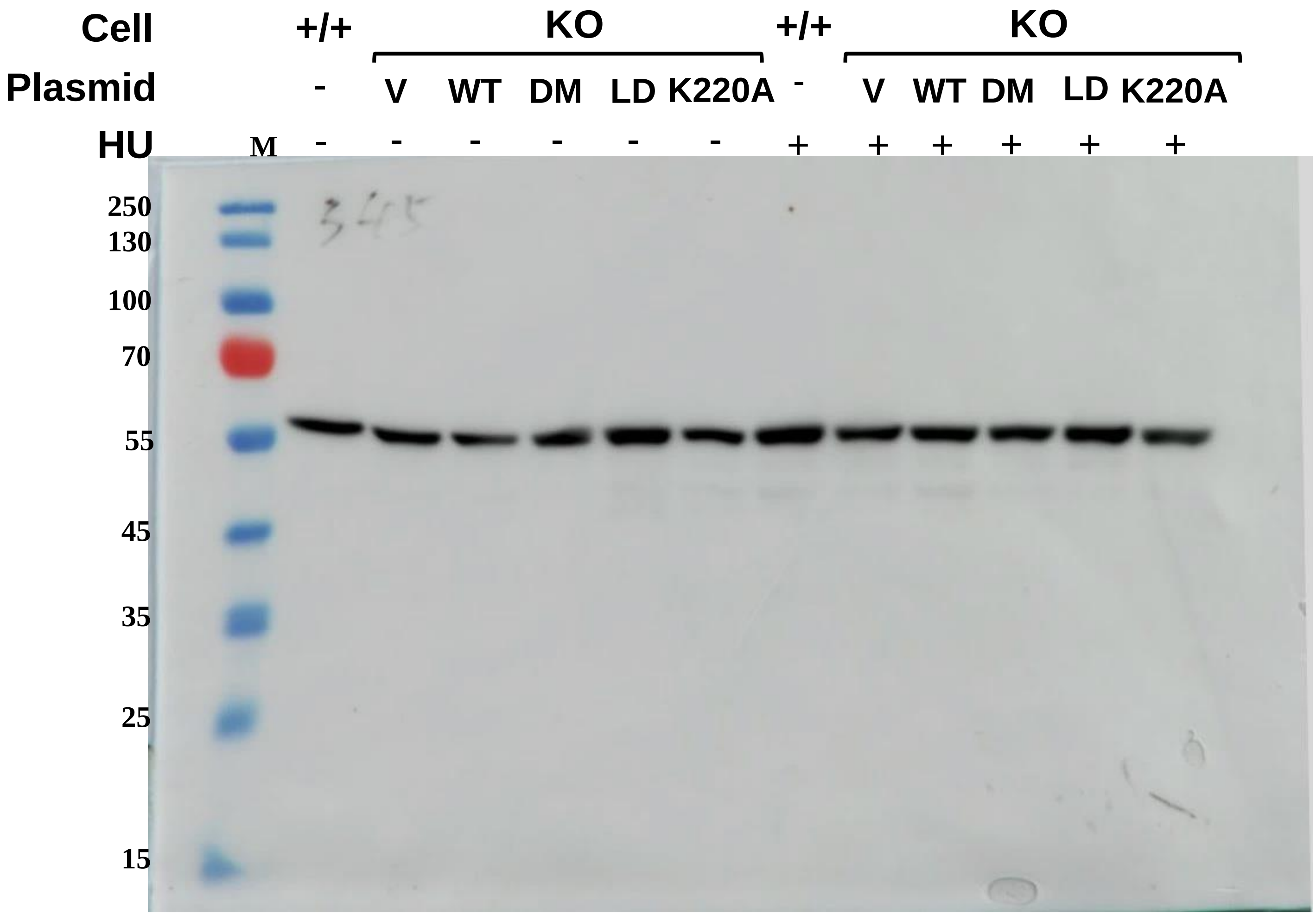

**Fig 6c beta actin loading control immunoblot. All lanes were used in this figure. PC3 Rad9 KO treated with 16 mM HU for 2 h and recovered for 2 h compared to no HU treatment.**

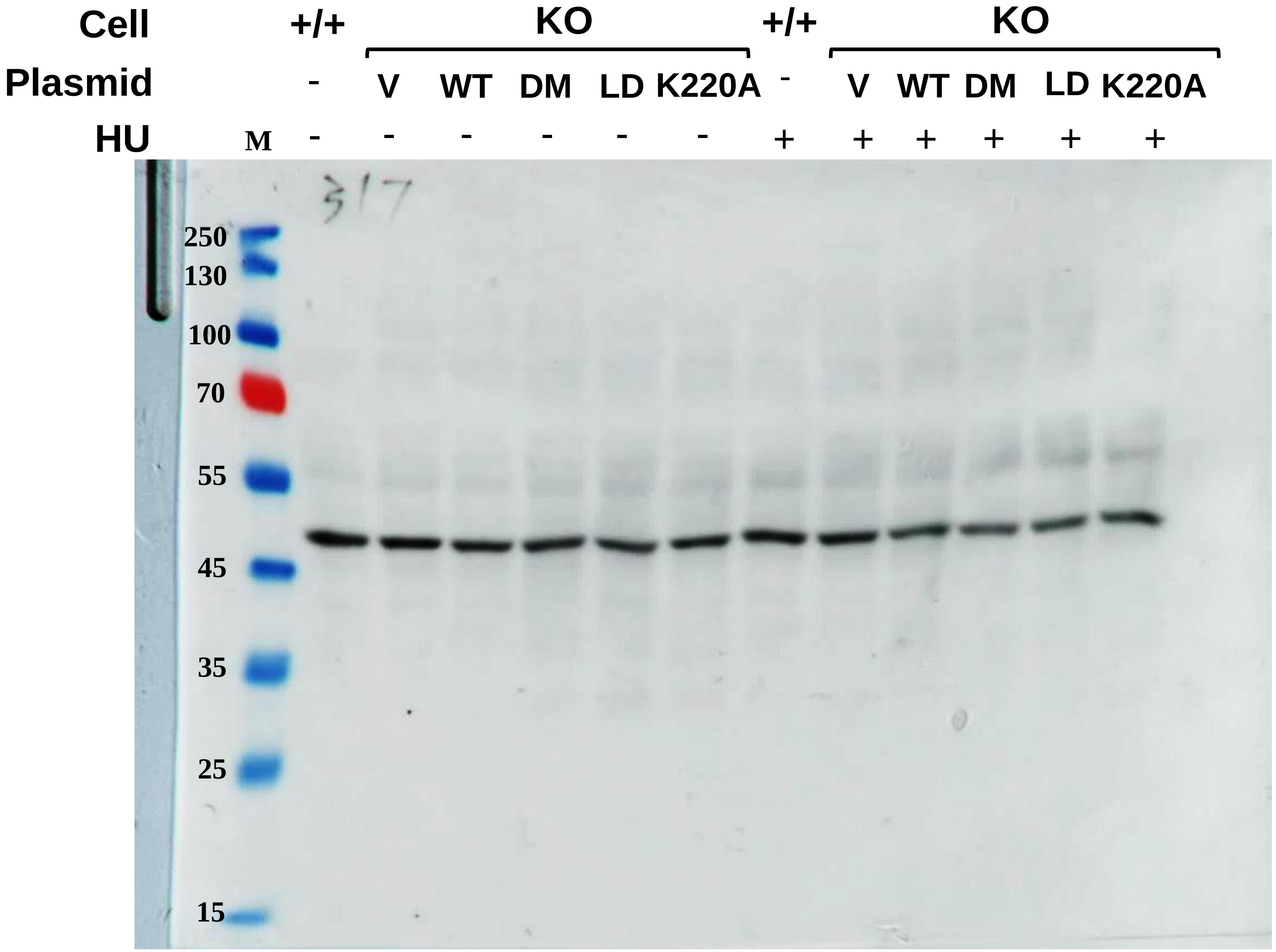

**Figure S1A and S1B full immunoblots: PC3 Rad9 KO treated with 16 mM HU for 2 h and recovered for 2 h. PC3 Rad9 KO cells treated with HU were used for blot images in supplemental figure 1.**

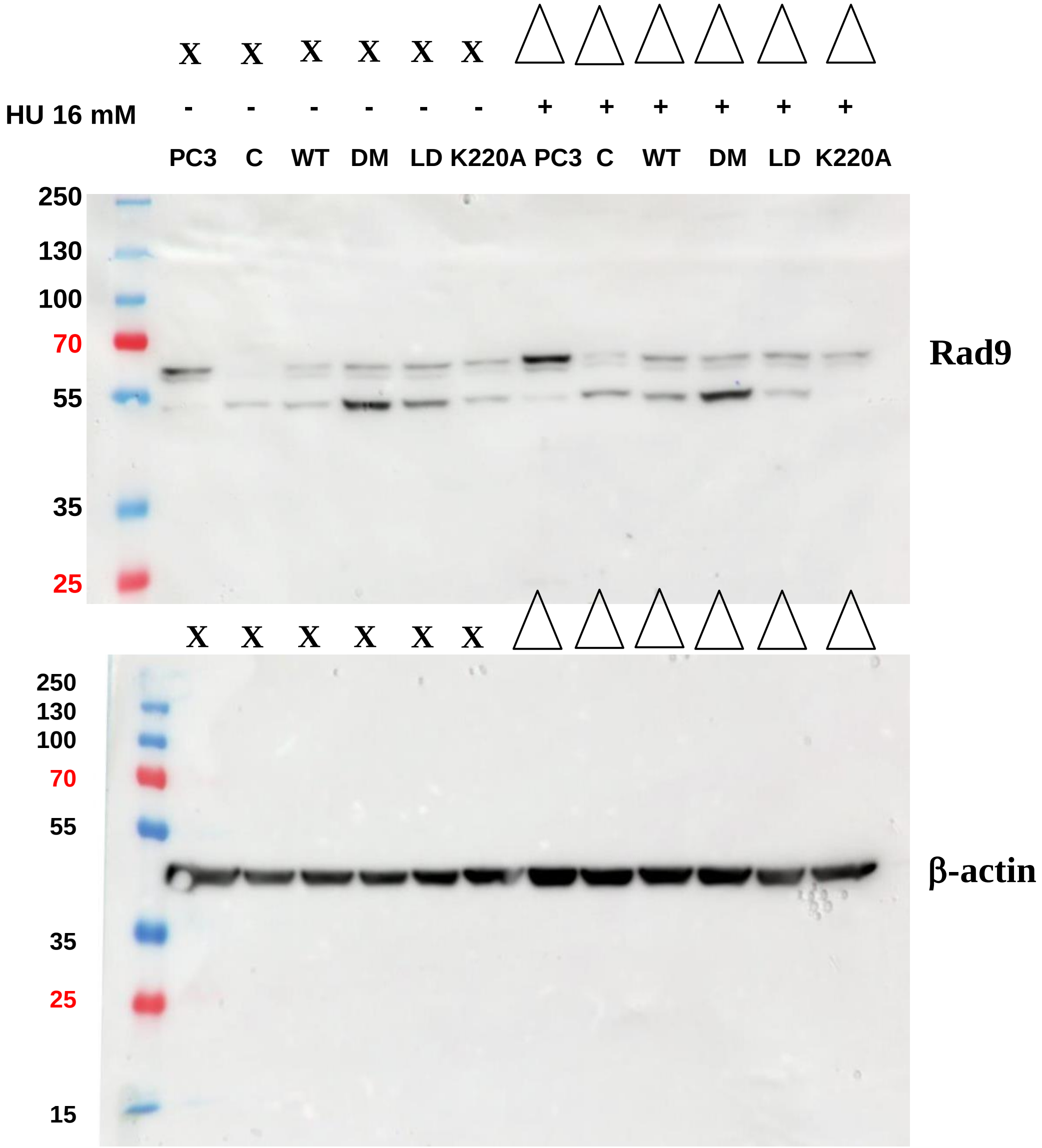

Supplement: S1 Raw images — (PDF) [file pone.0272645.s003.pdf]
